# Neuroimmune mechanisms of the cerebellum–brainstem network in autoimmune encephalitis and related neuroimmune disorders: antibody targets, selective vulnerability, and translational opportunities

**DOI:** 10.3389/fimmu.2026.1909595

**Published:** 2026-08-25

**Authors:** Yan Li, Yuyuan Hu, Yuhang Yang, Wenyuan Li, Minghua Fan, Zhijun Wang, Wei Zhang, Yanhong Wang

**Affiliations:** 1 Department of Neurology, Third Hospital of Shanxi Medical University, Shanxi Bethune Hospital, Shanxi Academy of Medical Sciences, Tongji Shanxi Hospital, Taiyuan, China; 2 Division of Colorectal Surgery, Third Hospital of Shanxi Medical University, Shanxi Bethune Hospital, Shanxi Academy of Medical Sciences, Tongji Shanxi Hospital, Taiyuan, China; 3 Yuncheng Central Hospital, Shanxi Medical University, Yuncheng, China

**Keywords:** autoimmune encephalitis, biomarkers, cerebellum–brainstem network, immune-mediated cerebellar ataxia, neuroimaging, neuronal surface antibodies, Purkinje cells, selective vulnerability

## Abstract

Autoimmune encephalitis (AE) comprises clinically and immunopathologically heterogeneous central nervous system disorders whose early recognition should be syndrome-based and should not depend on antibody results alone. Cerebellar and brainstem manifestations occur in selected AE and related central nervous system neuroimmune disorders, but their frequency, anatomical specificity, and mechanisms remain incompletely defined. In this narrative Review, we use the cerebellum–brainstem network as an anatomical and clinical organizing framework rather than proposing a discrete anatomical axis or a new disease entity. In selected neuronal-surface-antibody disorders, antibodies can directly alter receptor trafficking, receptor availability, protein interactions, or synaptic transmission, whereas intracellular-antigen-associated and paraneoplastic syndromes are more often linked to cytotoxic T-cell-dominant neuronal injury. To stabilize disease scope, central AE and directly relevant central nervous system autoimmune syndromes form the core evidence base; immune-mediated cerebellar ataxias, paraneoplastic syndromes, acute disseminated encephalomyelitis, and myelin oligodendrocyte glycoprotein antibody-associated disease are included only when they provide direct infratentorial evidence; and Miller Fisher syndrome and Guillain–Barré syndrome are retained solely as peripheral anatomical comparators, whereas Bickerstaff brainstem encephalitis represents a central brainstem syndrome. We present a hypothesis-generating circuit framework linking immune target engagement to cerebellar output and connected brainstem manifestations, while explicitly marking extrapolations from non-AE models as hypotheses [H]. We also distinguish a predominantly functional pattern from an established structural-injury pattern as non-sequential research constructs rather than stages, biomarker-defined transitions, treatment windows, or clinical algorithms. Current imaging studies demonstrate that infratentorial metabolic and structural abnormalities can occur, but no reproducible cerebellum–brainstem diagnostic, prognostic, or treatment-selection signature has been prospectively validated.

## 1 Introduction

Autoimmune encephalitis (AE) comprises heterogeneous inflammatory brain disorders whose early recognition should be based on the clinical syndrome, conventional investigations, and reasonable exclusion of alternative causes rather than on antibody results alone (1). Across AE subtypes, neuronal-surface antibodies, intracellular-antigen-associated cytotoxic T-cell responses, and secondary inflammatory processes can disrupt neural function through biologically distinct mechanisms (2, 3). Movement disorders, autonomic dysfunction, respiratory disturbance, and other extralimbic manifestations are recognized across antibody-defined syndromes (4), but these manifestations do not by themselves establish direct cerebellar or brainstem injury. The present Review therefore focuses on disorders with direct clinical, imaging, pathological, or mechanistic evidence relevant to infratentorial structures and explicitly separates such evidence from broader network-level or cross-disease inference.

The review is organized first by antigen localization and dominant immune effector mechanism, because directly pathogenic neuronal-surface antibodies and intracellular-antigen-associated, T-cell-dominant disorders differ in reversibility, tumor association, and risk of neuronal loss (2, 3). This mechanistic distinction is then mapped only to specific cerebellar or brainstem structures, circuits, phenotypes, and supporting evidence. Conventional MRI can be normal in clinically definite AE, but MRI negativity alone neither localizes dysfunction to the cerebellum or brainstem nor establishes a predominantly functional disease pattern; modality-specific imaging evidence is evaluated separately in Section 8.1.

Cerebellar and brainstem phenotypes require syndrome-specific anatomical localization because similar manifestations may arise from central autoimmune encephalitis, acquired demyelinating disease, paraneoplastic syndromes, or peripheral neuropathies. Within the anti-GQ1b spectrum, Bickerstaff brainstem encephalitis is retained as a central brainstem syndrome, whereas Miller Fisher syndrome is a predominantly peripheral Guillain–Barré-spectrum disorder included only as an anatomical boundary comparator; detailed anti-GQ1b mechanisms are discussed once in Section 4.3. Acute disseminated encephalomyelitis is an immune-mediated acquired central nervous system demyelinating syndrome (5), whereas MOG antibody-associated disease is a distinct MOG-IgG-associated demyelinating disorder in which brainstem or cerebellar presentations can occur (6); both are considered here only when direct infratentorial evidence is relevant.

This established distinction between directly antibody-mediated synaptic dysfunction and intracellular antigen-associated cytotoxic T-cell injury provides the mechanistic foundation on which the present cerebellum–brainstem anatomical framework is built (2, 3). Other immune-mediated disorders provide disease-specific examples of infratentorial involvement: GAD65 autoimmunity is a recognized cause of immune-mediated cerebellar ataxia, KLHL11-IgG is associated with paraneoplastic rhombencephalitis and cerebellar ataxia, and paraneoplastic cerebellar degeneration is characterized by immune-mediated cerebellar injury (7–10). Distinct disorders can produce overlapping cerebellar or brainstem phenotypes through different dominant mechanisms, including receptor or synaptic dysfunction in selected neuronal-surface-antibody diseases (2, 3), ganglioside-associated peripheral nerve injury in anti-GQ1b syndromes (11, 12), and cytotoxic T-cell-mediated neuronal injury in intracellular-antigen-associated paraneoplastic syndromes (3, 9); complement and glial mechanisms should be treated as disease-specific or hypothesis-generating rather than as components of a shared pathway [H] (13).

**Figure 1:**
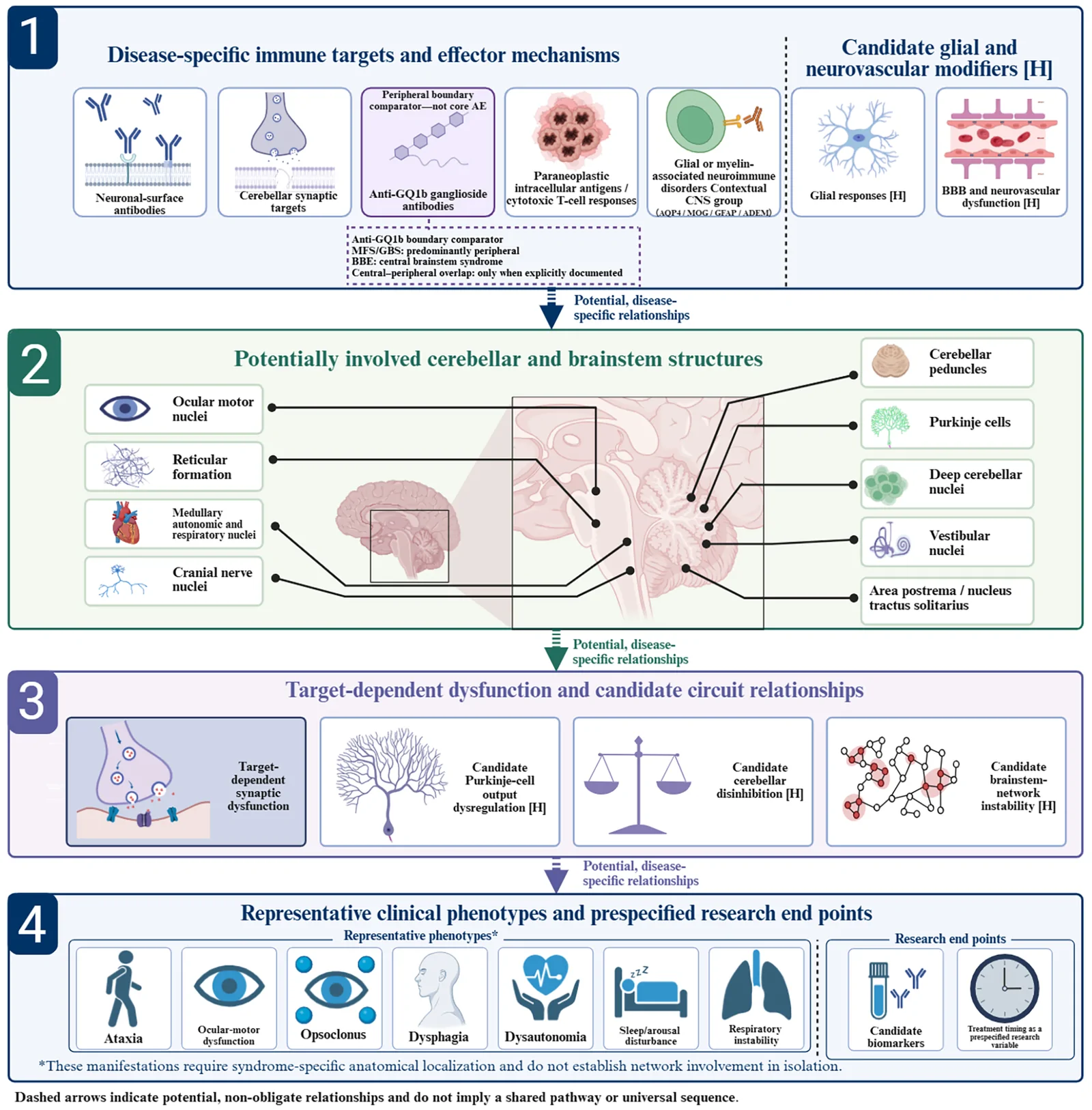
Hypothesis-generating framework of the cerebellum–brainstem network in autoimmune encephalitis and related neuroimmune disorders. This schematic presents the cerebellum–brainstem neuroimmune framework as a hypothesis-generating organization of disease-specific immune targets, effector mechanisms, candidate modifiers, potentially involved infratentorial structures, clinical phenotypes, and prespecified research end points. Disease-specific immune categories include neuronal-surface antibodies, cerebellar synaptic targets, intracellular-antigen-associated cytotoxic T-cell responses, and contextual glial or myelin-associated disorders; glial and neurovascular processes are shown separately as candidate modifiers [H]. The anti-GQ1b spectrum is depicted as an anatomical boundary comparator rather than core AE evidence: MFS and GBS are predominantly peripheral, BBE is a central brainstem syndrome, and central–peripheral overlap is reserved for explicitly documented overlap phenotypes. Potentially involved structures include Purkinje cells, deep cerebellar and vestibular nuclei, cerebellar peduncles, ocular-motor and cranial nerve nuclei, reticular and arousal systems, and medullary autonomic and respiratory networks; involvement of each structure must be established within the relevant disease. Target-dependent synaptic dysfunction is directly supported in selected neuronal-surface-antibody disorders, whereas proposed relationships involving Purkinje-cell output dysregulation, cerebellar disinhibition, and brainstem-network instability remain disease-specific hypotheses [H]. Representative manifestations include ataxia, ocular-motor dysfunction, opsoclonus, dysphagia, dysautonomia, sleep or arousal disturbance, and respiratory instability, but none establishes cerebellum–brainstem network involvement in isolation; candidate biomarkers and treatment timing are shown only as prespecified variables for prospective research. Dashed arrows indicate potential, non-obligate relationships and do not imply a shared pathway, universal sequence, treatment window, or validated clinical algorithm. AE, autoimmune encephalitis; BBB, blood–brain barrier; BBE, Bickerstaff brainstem encephalitis; GBS, Guillain–Barré syndrome; MFS, Miller Fisher syndrome; NTS, nucleus tractus solitarius; [H], hypothesis-generating inference.

In this Review, the term “cerebellum–brainstem network” refers to the interconnected infratentorial structures and circuits under discussion, whereas the “cerebellum–brainstem neuroimmune framework” refers to the hypothesis-generating organization of heterogeneous evidence; neither term denotes a new disease entity, a single projection pathway, or a unified immunopathological mechanism. Central AE and BBE constitute the principal CNS focus, whereas MFS and GBS are treated only as predominantly peripheral comparator disorders or as components of explicitly documented overlap syndromes (11, 12, 14). Based on established antibody- and T-cell-associated mechanisms (2, 3) and current evidence for immune-mediated cerebellar synaptopathy (15), **Figure 1** summarizes a four-tier, hypothesis-generating framework that organizes potential relationships among immune inputs, vulnerable infratentorial structures, circuit dysfunction, and clinical–translational research questions. This integrative and spatial framework distinguishes direct regional injury, secondary network dysfunction, and peripheral comparator mechanisms and generates explicit clinical, imaging, and biomarker questions for prospective testing rather than proposing a new immune pathway.

## 2 Literature search, eligibility, evidence appraisal, and scope of review

This article was prepared as a narrative Review rather than a formal systematic review. To improve methodological transparency and conceptual consistency, we specified the information sources, search period, eligibility criteria, disease-scope groups, and evidence-source categories used in the synthesis, consistent with the literature-search and evidence-appraisal domains emphasized by SANRA (16). The purpose was to provide a critical and hypothesis-generating integration of cerebellar and brainstem involvement across autoimmune encephalitis and related neuroimmune disorders rather than an exhaustive quantitative synthesis or a formal assessment of intervention effects.

PubMed/MEDLINE, Web of Science, Scopus, and Google Scholar were searched for publications available through 31 July 2026 using combinations of the following terms: “autoimmune encephalitis”, “cerebellar ataxia”, “brainstem encephalitis”, “rhombencephalitis”, “cerebellum–brainstem network”, “acute disseminated encephalomyelitis”, “ADEM”, “acquired demyelinating syndrome”, “myelin oligodendrocyte glycoprotein antibody-associated disease”, “MOGAD”, “anti-GQ1b”, “Bickerstaff brainstem encephalitis”, “Miller Fisher syndrome”, “paraneoplastic cerebellar degeneration”, “Purkinje cells”, “neuroinflammation”, “microglia”, “blood–brain barrier”, “cerebrospinal fluid biomarkers”, “neuroimaging”, “connectomics”, “resting-state functional MRI”, “spatial transcriptomics”, and “single-cell sequencing”.

Studies were eligible when they met at least one of the following criteria: (1) they examined antibody-defined or clinically defined autoimmune encephalitis with direct cerebellar, brainstem, or related infratentorial clinical, neuroimaging, neuropathological, biomarker, or mechanistic findings; (2) they examined a related central nervous system neuroimmune disorder that provided directly relevant evidence of cerebellar or brainstem involvement, including immune-mediated cerebellar ataxias, paraneoplastic neurological syndromes, GFAP-, AQP4-, or MOG-associated disease, or acute disseminated encephalomyelitis; or (3) they examined a predominantly peripheral disorder only when it provided an explicit anatomical comparator for distinguishing peripheral from central ocular-motor, vestibular, proprioceptive, or ataxic phenotypes. Consensus diagnostic criteria, disease-defining original studies, multicenter or large clinical cohorts, clinicopathological investigations, and disease-specific mechanistic studies were prioritized. Case series and case reports were retained only for rare syndromes or emerging immune targets for which higher-level evidence was unavailable and when they contributed a clearly defined anatomical, clinicopathological, or mechanistic observation.

We excluded general publications on autoimmune encephalitis or neuroinflammation that did not provide a direct contribution to cerebellar, brainstem, infratentorial-circuit, or disease-scope questions. Predominantly peripheral disorders were excluded unless they clarified a predefined peripheral–central anatomical boundary. Primary infectious encephalitis, vascular disease, neurodegenerative disease, and other non-autoimmune models were not included as core evidence; when such studies were required to generate a biological analogy, they were explicitly identified as indirect, hypothesis-generating evidence. Narrative reviews were used to identify and contextualize relevant literature but were not treated as substitutes for disease-defining original clinical, pathological, imaging, or experimental data.

For each included source, we extracted the disease or syndrome, anatomical compartment, immune target or effector mechanism, clinical phenotype, imaging or pathological correlate, study design, and directness of relevance to cerebellar or brainstem involvement. Interpretation considered both study design and evidentiary directness: a large study of general autoimmune encephalitis without infratentorial localization was not treated as direct cerebellum–brainstem evidence, whereas a smaller disease-specific clinicopathological study could provide more anatomically direct support for a narrowly defined claim.

The disease scope was organized into three explicitly defined groups. The core group comprised central autoimmune encephalitis and other antibody- or T-cell-mediated central nervous system syndromes with direct cerebellar or brainstem involvement. A contextual central nervous system group comprised immune-mediated cerebellar ataxias, paraneoplastic neurological syndromes, GFAP-, AQP4-, or MOG-associated disorders, and acute disseminated encephalomyelitis when their infratentorial findings informed anatomical localization, differential diagnosis, or mechanistic comparison. A peripheral boundary-comparator group comprised Miller Fisher syndrome, Guillain–Barré syndrome, and other predominantly peripheral anti-GQ1b phenotypes; these disorders were not classified as autoimmune encephalitis and were included only to distinguish peripheral ocular-motor or proprioceptive dysfunction from central Bickerstaff brainstem encephalitis or explicitly documented overlap syndromes. The aim was therefore to integrate convergent evidence without assuming that all included disorders share a common nosology, anatomical compartment, or pathogenic sequence.

Evidence was appraised according to source and directness rather than by a formal GRADE assessment, because this narrative Review did not address a single prespecified intervention question. We used four evidence-source categories: higher-directness human evidence, comprising consensus criteria, multicenter or large clinical cohorts, and directly relevant clinicopathological studies; supportive human evidence, comprising single-center cohorts, case series, case reports, and biomarker or neuroimaging associations; experimental evidence, comprising animal or *in-vitro* studies; and hypothesis-generating indirect inference, designated [H], comprising extrapolations from non-AE cohorts or other cross-disease models. Mechanistic statements were worded according to the highest directly supporting evidence category. Findings designated [H] were used to formulate testable questions and were not presented as established mechanisms of autoimmune encephalitis.

## 3 Neuroanatomical and functional basis of the cerebellum–brainstem network

The cerebellum and brainstem are anatomically distinct but functionally interconnected through sensorimotor, cerebellar, vestibular, ocular-motor, and autonomic pathways (17–19), providing an anatomical substrate for disease-specific hypotheses; direct synaptic or cellular immune involvement of individual structures must nevertheless be established within each disorder. The cerebellum supports prediction, timing, error-based adaptation, and coordinated sensorimotor control, whereas the brainstem contains cranial-nerve and vestibular nuclei, ascending and descending pathways, reticular systems, and cardiorespiratory and autonomic networks (17, 18, 20, 21). [H] This anatomical organization provides a basis for testing whether disease-specific cerebellar or brainstem involvement contributes to overlapping ocular-motor, bulbar, autonomic, respiratory, sleep-related, or cognitive manifestations in selected AE subtypes.

Reciprocal loops are central to the functional organization of the cerebellum–brainstem network (19). Cortical information reaches the cerebellum through pontine relays and mossy fiber pathways, while proprioceptive, vestibular, and visceral inputs enter through brainstem and spinal afferents. These inputs enter cerebellar cortical microcircuits through mossy- and climbing-fiber pathways; granule cells and inhibitory interneurons shape Purkinje-cell activity, and Purkinje cells provide the sole cortical output to the deep cerebellar and vestibular nuclei (19, 20). Purkinje-cell output regulates the deep cerebellar and vestibular nuclei, which communicate with thalamic, cortical, red-nuclear, reticular, ocular-motor, and spinal pathways (19). [H] Immune disturbance affecting one or more components of this loop—including synaptic receptors, ion channels, inhibitory interneurons, Purkinje cells, cerebellar nuclei, cerebellar peduncles, vestibular nuclei, or pontomedullary relays—could produce functionally related manifestations (7, 19), but this circuit-level inference requires disease-specific validation.

Ocular-motor manifestations provide a useful example of this organization. Eye movements require the integration of cerebellar gaze-holding mechanisms, vestibulo-ocular reflex modulation, saccadic calibration, pursuit control, and brainstem ocular-motor nuclei. In immune-mediated cerebellar syndromes, ocular-motor abnormalities such as opsoclonus, nystagmus, saccadic dysmetria, and impaired gaze stabilization can support infratentorial localization (7, 22), but they do not by themselves establish involvement of the entire cerebellum–brainstem network or identify a specific immune mechanism. In immune-mediated disease, opsoclonus–myoclonus–ataxia syndrome provides a clinically relevant model in which opsoclonus, myoclonus, ataxia, irritability, and sleep disturbance cluster within a single syndrome and are associated with an underlying neuroblastoma in approximately half of affected children (22). [H] In OMAS, proposed mechanisms of opsoclonus include hyperexcitability and reverberation within reciprocally connected saccadic burst-neuron circuits, with possible contributions from the cerebellar oculomotor vermis and fastigial nucleus; these circuit interpretations remain hypothesis-generating and should not be treated as established mechanisms of AE (22).

Bulbar and autonomic symptoms illustrate a similar principle. The medulla and pons contain densely organized nuclei and reticular circuits that coordinate swallowing, phonation, airway protection, respiration, cardiovascular regulation, arousal, and sleep–wake cycling. Classical lesion models such as lateral medullary syndrome demonstrate that disruption of a small brainstem territory can simultaneously produce vertigo, nystagmus, dysphagia, dysarthria, impaired gag reflex, ipsilateral ataxia, sensory deficits, and autonomic signs (23). Dysphagia in autoimmune neurological disease requires anatomical localization because dysfunction may arise from muscle, the neuromuscular junction, peripheral cranial nerves, the brainstem, or corticobulbar pathways (24).

Beyond motor coordination, cerebellar circuits contribute to cognitive and affective processing through distributed cerebello-cortical networks (25). Cerebellar microglia participate in synaptic remodeling, neuronal–glial communication, inflammatory responses, and maintenance of the local microenvironment in experimental and broader neurological contexts (13, 26); their disease-specific contribution to cerebellar or brainstem manifestations in AE remains unvalidated. [H] The possibility that regionally specialized cerebellar microglia translate intrinsic or systemic inflammatory signals into altered neuronal performance is supported by broader cerebellar and experimental literature (26), but has not been demonstrated in AE. Cerebellar dysfunction can affect motor coordination and cognitive–affective processing (25), but [H] the specific contribution of immune-mediated cerebellar dysfunction to fatigue, sleep disturbance, or behavioral change in AE remains unvalidated (7).

A clinically important feature of anti-NMDAR encephalitis is the discordance between severe functional impairment and conventional structural imaging: baseline MRI was normal in 28 of 53 patients (53%) in one clinical cohort and in 31 of 43 patients (72%) in a subsequent resting-state fMRI cohort (27, 28). Selected AE and related neuroimmune disorders can show direct infratentorial involvement: GABAB-receptor encephalitis is predominantly a limbic encephalitis, although cerebellar abnormalities have been reported in selected cases (29, 30); anti-IgLON5 disease has brainstem-dominant pathological involvement (31); and GFAP astrocytopathy can involve the brainstem and cerebellum as part of a broader central nervous system inflammatory syndrome (32). A neuroradiological perspective is therefore necessary but insufficient. A normal conventional MRI does not exclude AE, but it also does not by itself demonstrate cerebellum–brainstem network dysfunction; infratentorial localization requires concordant clinical findings and, where available, targeted metabolic, diffusion, volumetric, or functional imaging evidence (28, 33).

Accordingly, the cerebellum–brainstem network is considered here as a hypothesis-generating anatomical framework for investigating disease-specific regional vulnerability rather than as an established, uniformly immune-vulnerable unit. [H] The framework is intended to test whether target distribution and circuit anatomy contribute to overlapping cerebellar or brainstem manifestations across otherwise distinct disorders (2, 3, 7, 15); it does not imply that these disorders share a common pathogenic mechanism. Early gaze instability, opsoclonus, ataxia, dysphagia, sleep disturbance, respiratory irregularity, or autonomic instability should prompt syndrome-specific anatomical localization, but none of these manifestations alone establishes cerebellum–brainstem network involvement. The cerebellum–brainstem neuroimmune framework maps disease-specific antibody-mediated, cellular immune, and cerebellar synaptopathy mechanisms onto infratentorial anatomy, clinical phenotypes, and candidate research measures (2, 3, 15), without defining a shared pathway or treatment window.

## 4 Autoantibody and cellular targets relevant to the cerebellum–brainstem network

A mechanistic review of cerebellum–brainstem autoimmunity should first distinguish neuronal surface or synaptic targets that can be directly altered by antibodies from intracellular neuronal targets that more commonly identify cytotoxic T-cell-dominant disease, and should then consider how these established immune mechanisms map onto regional anatomy (2, 3). Antibody effects depend on both the target antigen and IgG subclass: IgG1-dominant antibodies such as NMDAR and AMPAR antibodies commonly induce receptor cross-linking or internalization, whereas IgG4-enriched antibodies such as LGI1 and CASPR2 antibodies more often disrupt protein–protein interactions; complement activation may contribute to selected disorders but is not a universal mechanism of neuronal surface-antibody disease (2, 3).

T-cell biology is also relevant to cerebellum–brainstem autoimmunity. CD8 T-cell cytotoxicity is particularly relevant to intracellular antigen-associated and paraneoplastic cerebellar or brainstem syndromes, in which neuropathological studies demonstrate close T-cell–neuron interactions and perforin–granzyme-mediated neuronal injury (2, 3, 34).

Preliminary HLA susceptibility data have been reported in KLHL11-IgG disease. In the Dubey et al. cohort, HLA-DQB1*02:01 and HLA-DRB1*03:01 were present in 7/10 and 6/10 genotyped patients, respectively (8); these findings require confirmation in larger cohorts and do not establish effects on chronicity or outcome. [H] Microglia–astrocyte–endothelial crosstalk and neurovascular-unit activation may amplify local immune injury through cytokine signaling, altered barrier permeability, complement activation, or glial-mediated synaptic remodeling (26, 35), but region-specific effects in cerebellar or brainstem AE have not been demonstrated. The cerebellum–brainstem neuroimmune framework is used to examine how antibody specificity, antigen localization, anatomical compartment, and immune effector biology may contribute to disease-specific infratentorial phenotypes, without assuming a shared mechanism or reversibility profile. Within this framework, antigen distribution, anatomical compartment, circuit organization, and local immune context are considered disease-specific variables rather than components of a shared pathogenic sequence. The framework therefore focuses on molecular targets and immune effector pathways affecting Purkinje cells, cerebellar interneurons, vestibular and ocular-motor pathways, pontomedullary circuits, cranial nerve nuclei, and autonomic regulatory networks (7, 15). [H] This organization generates testable hypotheses regarding why selected antibody-associated disorders may produce overlapping cerebellar, ocular-motor, bulbar, autonomic, respiratory, sleep-related, or cognitive–affective manifestations (15), but it does not establish a shared circuit mechanism. From a mechanistic perspective, an immunopathological classification may be more informative than anatomy alone. The major immunopathological categories relevant to the cerebellum–brainstem network are summarized in **Table 1**. Mechanistically, neuronal-surface-antibody AE and intracellular-onconeural disorders represent distinct central nervous system immunopathological categories (2, 3, 9), whereas the anti-GQ1b spectrum is a separate post-infectious ganglioside-associated group in which MFS and GBS phenotypes are predominantly peripheral, BBE is central, and explicitly defined overlap syndromes can involve both compartments (11, 12, 36).

**Table 1:** Disease-scope groups, representative immune mechanisms, anatomical compartments, infratentorial phenotypes, and evidence-source categories.

Category	Representative antibodies/targets	Main immune mechanism	Cerebellum–brainstem phenotype	MRI/biomarker features	Reversibility	Evidence-source category
Neuronal surface antibodies	NMDAR, AMPAR, GABAAR, GABABR, GlyR, mGluR1, LGI1, CASPR2, DPPX	Receptor cross-linking, internalization, functional blockade, altered excitatory–inhibitory balance, or disruption of synaptic adhesion and ion-channel organization	Encephalopathy, seizures, dyskinesia, autonomic instability, hypoventilation, dysphagia, ocular-motor disturbance, ataxia, or other cerebellar–brainstem signs	MRI may be normal or subtly abnormal early; functional or metabolic imaging may reveal network-level abnormalities; antibody titers and CSF findings may support immune activity	Variable and target-specific; reversibility cannot be inferred from neuronal-surface-antibody classification alone	Human cohort evidence for major AE subtypes; case series or case reports for rarer antibodies
Cerebellar synaptic targets and immune-mediated ataxias	GAD65, VGCC, GluRδ, Homer-3, EEF1D	Impaired inhibitory tone, altered calcium-dependent neurotransmission, Purkinje-cell synaptic dysfunction, impaired long-term depression, and reduced cerebellar reserve	Cerebellar ataxia, stiff-person spectrum, epilepsy, ocular-motor instability, dysarthria, tremor, and variable brainstem-predominant phenotypes	MRI may be normal early but may later show cerebellar atrophy; inflammatory CSF or neuronal injury markers may support active injury	Variable; early dysfunction may be modifiable, whereas established cerebellar atrophy is less reversible	Mainly case series and case reports for rare antigens; stronger mechanistic support for cerebellar synaptopathy
Peripheral anti-GQ1b boundary comparator—not core AE	GQ1b, GT1a, GD1b, GM1 and related ganglioside complexes	Post-infectious molecular mimicry; nodal, paranodal, nerve-terminal, or ganglioside-mediated dysfunction affecting ocular-motor and ataxic networks	Anatomical compartment and phenotype: predominantly peripheral in MFS and GBS-related phenotypes, with ophthalmoplegia, areflexia, sensory or proprioceptive ataxia, and peripheral weakness; central in BBE, with ophthalmoplegia and ataxia accompanied by impaired consciousness, hyperreflexia, or pyramidal signs; combined findings should be designated as central–peripheral overlap syndromes	Anti-GQ1b IgG supports diagnosis in many cases; MRI may be normal or variable; severe cases may show brainstem involvement or require intensive care support	Often favorable in uncomplicated MFS; severe BBE or GBS-overlap phenotypes may carry respiratory or autonomic risk, and the independent effect of immunotherapy on reversibility remains uncertain	Human clinical cohorts and case series; molecular mimicry supported by post-infectious association
Paraneoplastic intracellular antigens	Hu/ANNA1, Yo/PCA1, Ri/ANNA2, CV2/CRMP5, Ma2, amphiphysin, Tr/DNER, SOX1, KLHL11	Tumor-triggered cytotoxic T-cell-mediated neuronal injury; IFN-γ/pSTAT1 signaling, MHC-I upregulation, perforin–granzyme pathways, and neuronal apoptosis	Rapidly progressive cerebellar syndrome, rhombencephalitis, opsoclonus–myoclonus, dysarthria, dysphagia, dysautonomia, hearing or vestibular symptoms, and long-term disability	MRI may show cerebellar or brainstem involvement, atrophy, or may lag behind clinical decline; antibodies often act as diagnostic biomarkers rather than direct effectors	Often limited once neuronal loss occurs; stabilization depends on early tumor detection, oncological control, and immune treatment	Clinicopathological evidence and paraneoplastic cohort evidence; mechanism better established than many rare surface-antibody syndromes
Glial or myelin-associated neuroimmune disorders	AQP4, MOG, GFAP; ADEM as a clinical–radiological acquired demyelinating syndrome	Astrocytopathy, demyelination, complement activation, or cellular inflammation depending on target	Area postrema syndrome, medullary or brainstem syndrome, cerebellitis, opticomyelitis, dysphagia, nausea, hiccups, and variable cerebellar–brainstem involvement	MRI may show brainstem, cerebellar peduncle, spinal cord, optic pathway, or meningoencephalomyelitic involvement; CSF inflammation may be present	Variable; relapse prevention is critical, and reversibility depends on target, lesion burden, and treatment timing	Human cohort evidence for AQP4/MOG/GFAP disorders; relevance to AE-related cerebellum–brainstem involvement is contextual

Shared anti-GQ1b serology and post-infectious biology do not imply identical anatomical localization or inclusion of MFS/GBS within AE. MFS and GBS remain predominantly peripheral comparator disorders, BBE is a central brainstem syndrome, and combined involvement should be reserved for explicitly defined central–peripheral overlap phenotypes. Evidence-source categories comprise higher-directness human evidence, supportive human evidence, experimental evidence, and hypothesis-generating inference [H]. They describe source type and evidentiary directness and do not constitute a formal GRADE assessment.

### 4.1 Neuronal surface antibodies: target-dependent receptor and synaptic dysfunction

Neuronal surface and synaptic antibodies are clinically important because their extracellular epitopes are accessible to circulating or intrathecally produced IgG, allowing direct effects on receptor trafficking, receptor clustering, ligand interactions, ion-channel function, and synaptic organization (2, 3). In selected well-characterized neuronal-surface-antibody disorders, direct antibody effects can produce receptor, synaptic, or network dysfunction without immediate neuronal loss (2, 3, 37, 38), whereas the balance between functional disturbance and structural injury remains target- and disease-specific. This distinction is clinically relevant because similar cerebellar–brainstem phenotypes can reflect either predominantly functional synaptic disruption or destructive cellular immune injury, with different prognostic implications (2, 3).

Anti-NMDAR encephalitis, initially defined in young women with ovarian teratomas and subsequently recognized across ages, sexes, and tumor statuses, is the best-characterized neuronal surface-antibody encephalitis (38–41). Patient IgG targets an extracellular epitope on the GluN1 subunit and induces antibody-dependent cross-linking and internalization of surface and synaptic NMDARs, producing a titer-dependent and potentially reversible reduction in receptor density rather than immediate neuronal loss (2, 38, 40, 41). Anti-NMDAR encephalitis typically evolves through psychiatric or cognitive symptoms, seizures, speech dysfunction, movement abnormalities, reduced consciousness, autonomic instability, and central hypoventilation; these manifestations indicate dysfunction of distributed neural networks but do not by themselves demonstrate primary structural injury of the cerebellum or brainstem (38, 40–42). [H] Secondary alteration of cortico-ponto-cerebellar or cerebello-thalamo-cortical connectivity is considered a testable network-level inference rather than an established feature of anti-NMDAR encephalitis (41). AMPAR, GABAAR, and GABABR antibodies similarly perturb synaptic balance, but their phenotypes differ according to the excitatory or inhibitory receptor system affected. GABAAR encephalitis is characterized by severe seizures and multifocal cortical or subcortical brain lesions (43), whereas GABABR encephalitis predominantly presents with seizures and limbic encephalitis (29); cerebellar abnormalities in GABABR-associated disease should be regarded as selected case-based observations rather than a typical feature (30).

Among neuronal surface antibodies, mGluR1 is especially relevant to cerebellar neuroimmunology. mGluR1 is highly expressed at Purkinje-cell dendritic spines and participates in parallel fiber–Purkinje-cell signaling, long-term depression, vestibulo-cerebellar adaptation, motor learning, and maintenance of cerebellar internal models (15). Anti-mGluR1 encephalitis is therefore a relatively selective example of antibody-associated cerebellar circuit dysfunction. Patients with anti-mGluR1 encephalitis most often present with an acute or subacute cerebellar syndrome characterized by gait or limb ataxia, dysarthria, vertigo, nystagmus, diplopia, tremor, or dysmetria, with cognitive or behavioral manifestations reported in a subset of cases (44, 45). Early MRI may be normal, while later disease may show cerebellar T2/FLAIR abnormalities, leptomeningeal enhancement, or cerebellar atrophy (45). Serial imaging in anti-mGluR1 encephalitis has documented both initially normal scans and later cerebellar abnormalities, but the available case-based evidence does not establish a uniform synaptopathy-to-degeneration sequence (45).

GlyR-antibody-associated syndromes illustrate mechanistic and clinicopathological heterogeneity within surface-antibody neurological disease rather than a validated intermediate position between reversible synaptic dysfunction and persistent structural injury. Glycine receptors are widely expressed in the spinal cord and brainstem (46), where glycinergic inhibition contributes to motor control and respiratory rhythm generation (47). Anti-GlyR-antibody disease most commonly presents with progressive encephalomyelitis with rigidity and myoclonus or stiff-person-spectrum phenotypes and can also include seizures, encephalitis, cerebellar ataxia, dysautonomia, respiratory involvement, and other neurological manifestations (48). Proposed GlyR-antibody mechanisms include receptor internalization, functional blockade, and impaired inhibitory signaling. In a single PERM case with coexisting GlyR and GAD65 antibodies, neuropathology demonstrated CD8 T-cell inflammation, neuronal MHC-I upregulation, astrogliosis, microgliosis, and dentato-bulbar-spinal degeneration (49); this observation cannot establish the contribution of either antibody or be generalized to isolated GlyR-antibody disease.

### 4.2 Cerebellar synaptic targets and immune-mediated ataxias

This mechanistically heterogeneous group includes intracellular presynaptic antigens, neuronal-surface or membrane-associated cerebellar targets, and intracellular synaptic scaffolding proteins, whose pathogenic roles and clinical behavior differ substantially (7, 15). GAD65 is intracellular but concentrated at presynaptic inhibitory terminals, where it contributes to GABA synthesis, packaging, and release. Anti-GAD65-associated neurological syndromes include cerebellar ataxia, stiff-person spectrum disorder, epilepsy, and limbic encephalitis (7, 15). [H] In GAD65-associated cerebellar ataxia, impaired GABAergic transmission and altered Purkinje-cell firing have been proposed as candidate mechanisms (7, 15), but the direct pathogenic contribution of GAD65 antibodies remains incompletely established.

VGCC, mGluR1, and GluRδ antibodies also suggest that immune targeting of synaptic machinery may impair cerebellar computation before overt neuronal loss occurs. P/Q-type VGCCs regulate calcium-dependent neurotransmitter release, while mGluR1 and GluRδ are essential for long-term depression and plasticity at parallel fiber–Purkinje-cell synapses (15). Disruption of selected cerebellar synaptic targets can impair plasticity and motor adaptation (15, 19), whereas the direct antibody-mediated effect and evidentiary strength remain target-specific. This concept is relevant to autoimmune cerebellar ataxia, in which early dysfunction may reflect impaired synaptic learning or predictive coding rather than gross tissue destruction. Clinical reversibility depends on the antigen, immune effector mechanism, duration of active disease, and extent of established cerebellar injury, rather than on a uniform temporal sequence shared by all intracellular synaptic antigen-associated disorders (7, 15).

### 4.3 Anti-GQ1b antibody-associated disorders: peripheral MFS, central BBE, and defined overlap phenotypes

Anti-GQ1b antibody-associated disorders share post-infectious molecular mimicry but span distinct anatomical compartments, ranging from predominantly peripheral neuropathic phenotypes such as MFS and GBS overlap to the central brainstem syndrome of BBE (11, 12, 36). Anti-GQ1b IgG is strongly associated with MFS and is also detected in acute ophthalmoplegia, selected GBS overlap syndromes, and BBE, supporting a shared immunological spectrum while preserving the nosological distinction between predominantly peripheral and central phenotypes (11, 12, 36). In MFS, electrophysiological and anatomical evidence supports predominant involvement of peripheral ocular-motor nerves and proprioceptive or muscle-spindle afferents, whereas impaired consciousness, hyperreflexia, or pyramidal signs identify central brainstem involvement in BBE (12, 14, 50, 51).

The pathogenesis is typically post-infectious. Molecular mimicry between microbial lipooligosaccharides and host gangliosides can induce IgG antibodies that bind GQ1b, GT1a, GD1b, or related ganglioside complexes (52). In Miller Fisher syndrome, this mechanism primarily affects peripheral ocular-motor and proprioceptive pathways (11, 51). BBE should be regarded as a central post-infectious brainstem syndrome defined by ophthalmoplegia and ataxia together with impaired consciousness or pyramidal signs, although peripheral nerve involvement may coexist in BBE–GBS overlap cases (12, 14). Importantly, anti-GQ1b antibody negativity does not exclude Bickerstaff brainstem encephalitis, because diagnosis remains grounded in the central brainstem clinical syndrome rather than in a single serological result (14, 53). Uncomplicated MFS usually has a favorable natural history, whereas severe BBE or GBS-overlap phenotypes may require intensive supportive care (11, 12); evidence that immunotherapy independently determines reversibility remains limited. Early recognition remains important, but the source of risk should be specified: uncomplicated MFS is usually a peripheral and self-limited neuropathic syndrome, whereas ventilatory failure, severe bulbar dysfunction, or intensive-care dependency should raise concern for GBS overlap or severe central BBE (11, 14).

### 4.4 Paraneoplastic intracellular antigens: tumor immunity, CD8 T cells, and neuronal injury

Paraneoplastic neurological syndromes involving the cerebellum or brainstem should be classified according to the updated PNS-Care criteria, which integrate the neurological phenotype, antibody cancer-risk category, presence of cancer, and follow-up information to assign possible, probable, or definite PNS (9). Antibodies such as Hu/ANNA1, Yo/PCA1, Ri/ANNA2, CV2/CRMP5, Ma2, amphiphysin, Tr/DNER, SOX1, and KLHL11 should be interpreted within their established cancer-risk categories and in relation to the neurological phenotype and oncological context rather than regarded as stand-alone evidence of PNS (9). In intracellular antigen-associated PNS, the antibodies primarily function as diagnostic biomarkers, whereas neuronal injury is more strongly linked to antigen-specific cytotoxic T-cell responses (2, 3). The predominant effector mechanism involves cytotoxic T cells directed against shared tumor–neural antigens, with neuropathological evidence supporting close T-cell–neuron interactions and perforin–granzyme-mediated neuronal degeneration (2, 3).

This mechanism may account for the severe prognosis of many paraneoplastic cerebellar and brainstem syndromes. Anti-Yo/PCA1 is strongly associated with rapidly progressive paraneoplastic cerebellar degeneration and Purkinje-cell injury. Anti-Hu/ANNA1 is associated with encephalomyelitis and sensory neuronopathy and may include autonomic or brainstem involvement, whereas anti-Ri/ANNA2 is classically linked to opsoclonus–myoclonus–ataxia. CV2/CRMP5-associated paraneoplastic neurological syndromes can involve both central and peripheral nervous system compartments, including cerebellar, brainstem, basal-ganglia, optic, myelopathic, or neuropathic phenotypes (54). Amphiphysin may produce stiff-person spectrum disorder, encephalomyelitis, and cerebellar involvement (10, 34, 55). In intracellular-antigen-associated PNS, these clinical signs may reflect cellular immune-mediated neuronal injury rather than direct antibody-mediated receptor dysfunction (2, 3, 9), but the extent and reversibility of injury remain disease- and patient-specific.

KLHL11-IgG-associated disease provides a documented example of paraneoplastic, T-cell-linked brainstem–cerebellar autoimmunity associated with rhombencephalitis, cerebellar ataxia, vestibulocochlear symptoms, and germ-cell or other tumors; its imaging, neuropathological, and antigen-specific cellular findings are reported in detail in Section 8.1 (8).

Extracellular target accessibility helps determine whether antibodies can directly alter neuronal surface or synaptic proteins (2, 3); potential blood–brain barrier and regional neurovascular modifiers are considered separately in Section 6 [H] (35). In intracellular antigen-associated PNS, immune-cell phenotype and established tissue injury are major determinants of reversibility, whereas treatment-timing effects should be evaluated within disease-specific cohorts rather than inferred from MRI findings (9, 10, 34).

## 5 Disease-specific circuit hypotheses within the cerebellum–brainstem network

**Figure 2:**
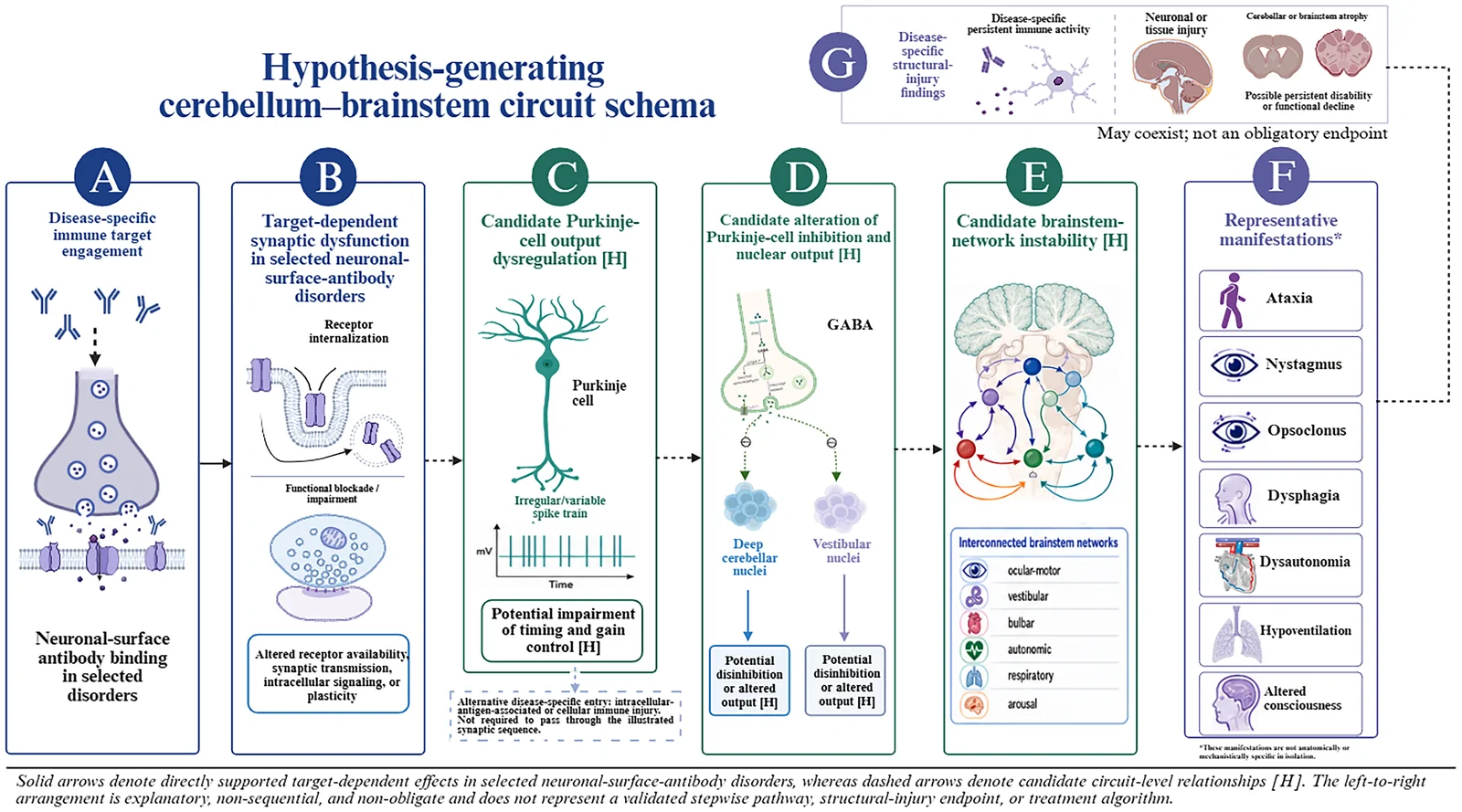
Hypothesis-generating circuit schema for disease-specific cerebellum–brainstem dysfunction. This schematic distinguishes directly supported target-dependent mechanisms in selected neuronal-surface-antibody disorders from candidate circuit-level relationships involving Purkinje-cell output, cerebellar nuclear signaling, and connected brainstem networks [H]; the A–G arrangement is explanatory, non-sequential, and non-obligate. In selected neuronal-surface-antibody disorders, antibody binding may directly alter receptor or synaptic function, whereas intracellular-antigen-associated and other cellular immune disorders enter through separate disease-specific mechanisms and are not required to pass through the illustrated synaptic sequence. Target-dependent effects can alter receptor availability, synaptic transmission, intracellular signaling, or plasticity in selected disorders; proposed relationships with Purkinje-cell firing, cerebellar nuclear output, and downstream brainstem function remain hypotheses requiring disease-specific validation [H]. [H] Altered Purkinje-cell inhibitory output could modify deep cerebellar or vestibular nuclear signaling and contribute to dysfunction within connected ocular-motor, vestibular, bulbar, autonomic, respiratory, or arousal-related brainstem networks, but this relationship has not been validated across AE subtypes. Representative manifestations include ataxia, nystagmus, opsoclonus, dysphagia, dysautonomia, hypoventilation, and altered consciousness, but these findings do not establish a common circuit mechanism or anatomical localization in isolation. Disease-specific persistent immune activity or cellular immune injury may coexist with neuronal loss, axonal or tissue injury, cerebellar or brainstem atrophy, and long-term disability; these findings are shown separately and are not obligatory endpoints of the candidate circuit relationships. Solid arrows denote directly supported target-dependent effects in selected neuronal-surface-antibody disorders, whereas dashed arrows denote candidate circuit-level relationships [H]. Peripheral anti-GQ1b mechanisms are not represented as a CNS circuit sequence. AE, autoimmune encephalitis; [H], hypothesis-generating inference.

A central unresolved question is how disease-specific immune targets and effector mechanisms contribute to overlapping cerebellar or brainstem phenotypes across autoimmune encephalitis and related neuroimmune disorders (2, 3, 9). Within the cerebellum–brainstem network, this question is relevant because selected immune-mediated disorders can combine cerebellar, ocular-motor, bulbar, autonomic, sleep-related, or arousal-related manifestations. The schema focuses on how molecularly specific synaptic perturbations may alter cerebellar output and connected brainstem networks; the primary imaging evidence is reviewed separately in Section 8.1. To translate established synaptic and cerebellar mechanisms into testable circuit-level questions, we present a non-linear schema that organizes disease-specific relationships among immune target engagement, synaptic dysfunction, Purkinje-cell output, cerebellar nuclear signaling, connected brainstem networks, clinical manifestations, and structural injury (**Figure 2**) (2, 3, 15). These components may occur in parallel, in different orders, or be absent in individual antibody-defined or cellular immune-mediated disorders; their arrangement does not define a new or obligatory pathogenic sequence (2, 3, 7, 15).

At the molecular–synaptic level, disease-specific antibodies or cellular immune responses may engage functionally relevant neuronal or glial targets. In neuronal surface-antibody syndromes, antibodies bind native extracellular epitopes on receptors, ion channels, or synaptic adhesion proteins. These antibodies are often associated initially with altered synaptic transmission rather than immediate neuronal death (56). Similar principles may apply to AMPAR, GABAAR, GABABR, LGI1, CASPR2, GlyR, DPPX, and mGluR1 antibodies. However, their downstream effects differ according to whether the targeted system supports excitatory drive, inhibitory tone, channel clustering, neurotransmitter release, or synaptic plasticity (56, 57). In selected neuronal-surface-antibody disorders with direct mechanistic evidence, early dysfunction can reflect target-dependent synaptic disturbance rather than overt neuronal destruction (2, 3).

Within this molecular–synaptic level, receptor internalization, functional blockade, or disruption of synaptic organization may occur in a target-dependent manner (2, 3). Depending on the target, epitope, and local circuit context, antibody-driven reduction of surface receptors may diminish physiological neurotransmission. By contrast, antibodies interfering with inhibitory receptors may promote disinhibition and hyperexcitability. Electrophysiological studies of antibody-associated encephalitis suggest that changes in neuronal excitability, synaptic strength, and plasticity may help account for both hyperexcitability-related manifestations, such as seizures or dyskinesias, and loss-of-function phenotypes, such as cognitive impairment, ataxia, or autonomic dysregulation (57). In the cerebellum, this distinction is especially important. Motor and ocular-motor coordination depend not only on the magnitude of excitation or inhibition, but also on the timing of climbing fiber error signals, parallel fiber input, inhibitory interneuron modulation, and Purkinje-cell firing regularity (58).

At the cerebellar-output level, Purkinje-cell firing and inhibitory control may be disrupted. Purkinje-cell inhibitory output regulates deep cerebellar and vestibular nuclear activity relevant to motor, ocular-motor, vestibular, and cognitive–affective processing (19). Single-nucleus transcriptomic work further indicates that Purkinje cells are not a uniform population. Aldoc-positive and Plcb4-positive Purkinje neurons have distinct molecular signatures, physiological properties, and learning-related plasticity (59). [H] Purkinje-cell transcriptional heterogeneity supports the testable hypothesis that immune perturbation may differentially affect cerebellar modules and contribute to variation in gait, ocular-motor, cognitive–affective, or autonomic phenotypes; this inference has not been validated in AE.

[H] Experimental models of cerebellar inflammation show altered Purkinje-cell excitability, synaptic plasticity, and afferent organization (58), but these findings have not been validated in cerebellar or brainstem AE. [H] Irregular Purkinje-cell firing, reduced inhibitory output, or structural Purkinje-cell loss could alter deep cerebellar and vestibular nuclear signaling and thereby contribute to ataxia or related circuit dysfunction (19), but this causal sequence has not been directly validated in AE.

At the connected brainstem-network level, disease-specific dysfunction may involve vestibular, ocular-motor, bulbar, autonomic, respiratory, or arousal-related systems. [H] Through established projections from the deep cerebellar and vestibular nuclei (17, 19), altered cerebellar output could contribute to instability in ocular-motor, vestibular, reticular, autonomic, or respiratory brainstem networks, but this causal relationship has not been demonstrated in AE. [H] Candidate downstream manifestations may include impaired gaze holding or vestibulo-ocular adaptation (19), altered vestibulo-autonomic integration (17), swallowing or visceral-autonomic dysregulation (60), and respiratory instability (21), but a common AE-specific circuit pattern has not been established. [H] OMAS is retained only as a non-AE comparator for generating ocular-motor and cerebellar–brainstem circuit hypotheses (22); its proposed vermis–fastigial and saccadic burst-neuron mechanisms cannot be assumed to apply directly to AE. [H] Because brainstem nuclei are densely interconnected, future studies should test whether altered cerebellar timing contributes to clinically prominent brainstem manifestations rather than assuming such a relationship.

Autonomic instability warrants particular attention because it can be a life-threatening manifestation of selected AE subtypes, including anti-NMDAR encephalitis (40, 41), although its anatomical and circuit-level mechanisms remain disease-specific. Brainstem autonomic circuits, including the nucleus tractus solitarius and ventrolateral medullary networks together with hypothalamic projections, regulate integrated sympathetic, parasympathetic, and respiratory output (21, 60). Autonomic instability and central hypoventilation are established manifestations of anti-NMDAR encephalitis, whereas a causal role for microglial remodeling within specific brainstem autonomic nuclei remains unvalidated (38, 40).

Structural injury is considered separately as a disease-specific pattern relevant particularly to selected prolonged, relapsing, intracellular-antigen-associated, or paraneoplastic disorders (2, 3, 9), rather than as the final or obligatory consequence of neuronal-surface-antibody disease. Structural injury is disease-specific: cytotoxic T-cell responses are particularly relevant to intracellular-antigen-associated and paraneoplastic disorders (3, 9), whereas direct receptor or synaptic dysfunction is better established in selected neuronal-surface-antibody diseases (2). Complement and glial mechanisms remain disease-dependent or hypothesis-generating rather than obligatory endpoints.

The schema is intended to generate disease-specific mechanistic, imaging, and biomarker hypotheses and does not define treatment timing or a therapeutic window.

## 6 Candidate determinants of cerebellar and brainstem vulnerability

Current evidence does not establish a single mechanism of selective cerebellar or brainstem vulnerability across AE; instead, disease-specific observations support several candidate determinants that require prospective validation. These candidate determinants include target-antigen distribution, neuronal and synaptic physiology, neurovascular context, and local glial–immune interactions, but their relative contributions are likely to differ across disorders. Distinct antibodies may target different proteins, yet they may produce overlapping phenotypes when these targets are enriched in Purkinje-cell networks, vestibular nuclei, ocular-motor pathways, medullary autonomic circuits, or brainstem arousal systems.

First, selective vulnerability is shaped by antigen distribution. The cerebellum contains highly specialized neurons and synaptic scaffolds that are frequent targets in immune-mediated ataxias. Homer-3 is a postsynaptic-density scaffold enriched in Purkinje cells that colocalizes with and modulates group I metabotropic glutamate receptors (61). Homer-3 autoimmunity provides a case-based example of an antigen-associated cerebellar syndrome that may resemble degenerative ataxia, but current evidence neither establishes a general rule of cerebellar susceptibility nor permits firm conclusions regarding treatment responsiveness (61).

Second, Purkinje cells exhibit high-frequency firing, extensive parallel-fiber integration, and calcium-dependent synaptic plasticity (19); [H] whether these physiological properties increase susceptibility to immune injury in AE remains unvalidated.

Third, regional barrier and neurovascular properties may act as permissive modifiers of immune access, although their contribution to anatomically patterned AE remains hypothesis-generating [H] (35). The blood–brain barrier is formed primarily by specialized CNS endothelial cells and their tight junctions, with pericytes, astrocytic endfeet, basement membrane, and other neurovascular-unit cells contributing to barrier maintenance and neurovascular signaling (35, 62, 63). Peripheral inflammation may alter tight junction integrity, endothelial adhesion molecule expression, cytokine signaling, and immune-cell trafficking. [H] Experimental and transcriptomic studies indicate that blood–brain barrier and neurovascular-unit properties vary across CNS regions (64, 65), but whether such regional specialization modifies cerebellar or brainstem susceptibility in AE remains untested. In neuroimmunological disease, BBB disruption can permit entry of pathogenic antibodies or lymphocytes into the CNS. Thus, barrier dysfunction should be considered not as a generic explanation for infratentorial involvement, but as a permissive mechanism. [H] Barrier dysfunction could interact with antigen distribution and local circuit properties to modify regional immune exposure (35), but preferential cerebellar or brainstem access has not been demonstrated.

Fourth, [H] Purkinje cells, dentate neurons, vestibular nuclei, medullary autonomic neurons, and cranial motor nuclei occupy distinct glial and neurovascular environments, and broader experimental evidence indicates that microglia, astrocytes, and neurovascular-unit cells can modify inflammatory signaling, synaptic remodeling, debris clearance, and repair (13, 35); whether such regional differences determine cerebellar or brainstem phenotypes in AE remains unvalidated.

The area postrema and nucleus tractus solitarius form a dorsal medullary interface for circulating and vagal visceral signals (60, 66), whereas vestibulo-autonomic and respiratory integration is mediated through partially distinct brainstem networks (17, 21). [H] The anatomical position and visceral connections of the area postrema and nucleus tractus solitarius support testing whether barrier-related or visceral immune perturbations contribute to autonomic, respiratory, or arousal-related manifestations; however, these symptoms and limited conventional MRI abnormalities do not by themselves establish regional immune injury (35, 60).

Neuropathological studies of autoimmune ataxias show that inflammatory infiltrates are most conspicuous in some paraneoplastic forms, whereas other cases may show limited inflammation with prominent Purkinje-cell loss and pathology concentrated in the cerebellum and its connections; this pattern can mimic hereditary or sporadic degenerative ataxia (10, 67). This distinction is clinically important. Across these four candidate dimensions, Purkinje-cell loss and cerebellar atrophy have been documented in selected autoimmune and paraneoplastic cerebellar disorders, but available evidence does not establish a uniform progression from a predominantly functional pattern to structural injury (8, 67, 68).

The proposed vulnerability framework therefore considers antigen distribution, neuronal and synaptic physiology, barrier properties, and local glial–vascular context as candidate, disease-specific determinants rather than components of a validated common pathway (7, 15). Clinically, cerebellar or brainstem symptoms in AE—particularly ataxia, gaze instability, dysarthria, dysphagia, sleep disturbance, or dysautonomia—should prompt circuit-informed localization and syndrome-specific evaluation rather than be interpreted as evidence of a single anatomical lesion (1, 7, 69). The proposed selective-vulnerability framework comprises four interacting dimensions: antigen-rich synapses, physiologically demanding neurons, heterogeneous barrier interfaces, and regionally distinct glial–vascular niches. The major biological dimensions contributing to this selective vulnerability are summarized in **Table 2**.

**Table 2:** Candidate determinants of cerebellar and brainstem vulnerability and their evidentiary boundaries.

Vulnerability dimension	Main structures/cells	Mechanistic explanation	Representative examples	Evidence strength	Clinical implication
Antigen enrichment	Purkinje cells, cerebellar interneurons, deep cerebellar nuclei, vestibular nuclei, ocular-motor pathways	Immune targets enriched in specific cerebellar or brainstem circuits may produce anatomically patterned phenotypes despite heterogeneous antibody specificity	mGluR1, GAD65, Homer-3, Zic4, KLHL11, Tr/DNER	Human case series and case reports; mechanistic support from antigen localization and cerebellar biology	Helps explain why different antibodies may converge on ataxia, ocular-motor instability, dysarthria, or vestibular symptoms
Neuronal and synaptic physiology	Purkinje cells, selected brainstem autonomic nuclei, vestibular nuclei, and reticular systems	High-frequency firing, calcium-dependent plasticity, long-range projections, and continuous neuromodulatory activity may increase sensitivity to inflammatory or synaptic stress	Purkinje-cell firing and synaptic-plasticity properties; brainstem physiological demands as hypothesis-generating considerations [H]	Strong biological plausibility; direct AE-specific validation remains limited	May explain disproportionate symptoms despite subtle MRI abnormalities and may define circuits vulnerable to relapse or chronic injury
Barrier heterogeneity	Blood–brain barrier, blood–CSF barrier, area postrema, neurovascular unit, perivascular immune niches	Regional differences in permeability, endothelial activation, cytokine signaling, and immune-cell trafficking may permit antibody or cellular immune access to selected CNS compartments	BBB disruption, area postrema involvement, medullary autonomic nuclei, cerebellar peduncle involvement	Experimental or broader neuroimmunological evidence; application to cerebellar or brainstem AE remains hypothesis-generating [H]	Supports interpreting BBB dysfunction as a permissive modifier rather than a nonspecific explanation
Glial–vascular niche	Microglia, astrocytes, endothelial cells, pericytes, perivascular immune cells	Local glial and vascular responses may amplify or buffer immune injury through cytokines, complement signaling, oxidative stress, synaptic pruning, and debris clearance	Microglia–astrocyte–endothelial crosstalk; complement-mediated synaptic remodeling; cytokine amplification	Experimental or broader neuroimmunological evidence; application to cerebellar or brainstem AE remains hypothesis-generating [H]	May modify local inflammatory, synaptic-remodeling, and repair responses, but does not define a validated transition from functional dysfunction to structural injury [H]
Circuit-level convergence	Deep cerebellar nuclei, vestibular nuclei, ocular-motor nuclei, reticular formation, medullary autonomic nuclei	Distinct immune mechanisms may converge on shared output pathways, producing overlapping motor, ocular, bulbar, autonomic, respiratory, and arousal-related symptoms	Ataxia, nystagmus, opsoclonus, dysphagia, dysautonomia, hypoventilation, altered consciousness	Strong anatomical and functional rationale; clinical validation requires standardized phenotyping	Supports circuit-informed clinical assessment rather than lesion-based interpretation alone

## 7 Two non-sequential research patterns: predominantly functional dysfunction and established structural injury

**Figure 3:**
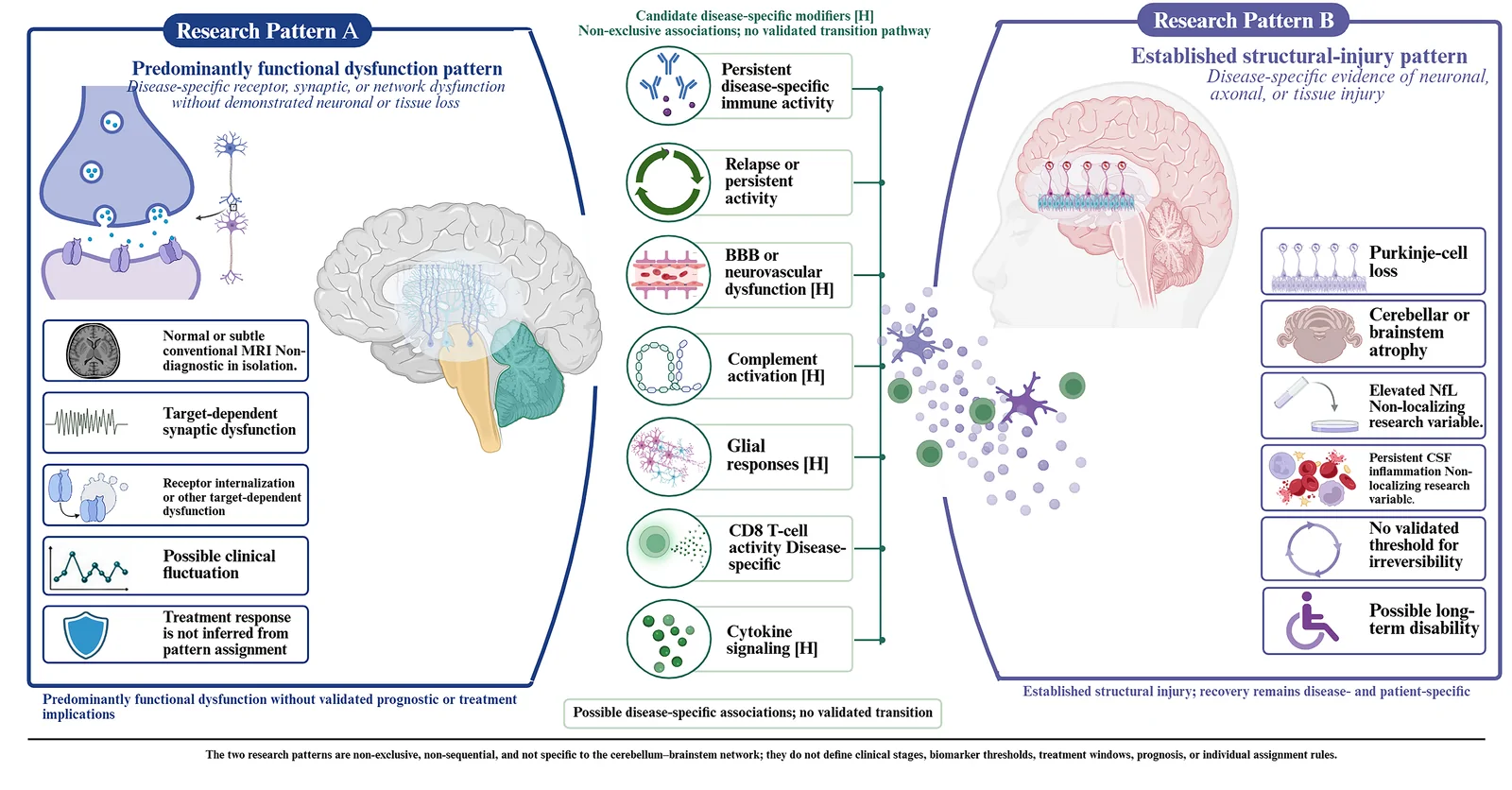
Two non-exclusive, non-sequential research patterns of predominantly functional dysfunction and established structural injury. This schematic distinguishes two non-exclusive research patterns—predominantly functional dysfunction and established structural injury—without defining a universal temporal sequence, clinical staging system, or transition pathway. The predominantly functional dysfunction pattern describes disease-specific receptor, synaptic, or network dysfunction without demonstrated neuronal or tissue loss and may include target-dependent receptor dysfunction, clinical fluctuation, and normal or subtle conventional MRI findings; however, MRI negativity does not establish this pattern, and the pattern does not independently predict reversibility or treatment response. Candidate disease-specific modifiers include persistent immune activity, relapse, BBB or neurovascular dysfunction, complement and glial responses [H], CD8 T-cell activity, and cytokine signaling; these factors are non-exclusive associations and do not constitute a validated transition pathway between the two research patterns. The established structural-injury pattern describes disease-specific evidence of neuronal, axonal, or tissue injury, including neuropathological neuronal loss or longitudinally documented cerebellar or brainstem atrophy; elevated NfL and persistent CSF inflammation are shown only as non-localizing candidate research variables and do not define this pattern or irreversibility. The two patterns may coexist, are not specific to the cerebellum–brainstem network, and do not define clinical stages, biomarker thresholds, prognosis, treatment windows, or individual treatment assignment; treatment timing remains a prespecified variable for prospective evaluation within disease- and mechanism-defined cohorts. BBB, blood–brain barrier; CSF, cerebrospinal fluid; MRI, magnetic resonance imaging; NfL, neurofilament light chain; [H], hypothesis-generating inference.

To organize heterogeneous evidence without implying a universal disease sequence, we distinguish two non-exclusive research patterns—predominantly functional dysfunction and established structural injury (**Figure 3**). The framework is intended for prospective testing, is not specific to the cerebellum–brainstem network, and does not constitute clinical staging, an obligatory transition model, a biomarker-defined threshold, or a therapeutic window. Persistent inflammation, relapse, and cellular immune activation may be associated with structural injury in selected disorders (3, 9). [H] Glial amplification is considered a disease-dependent candidate modifier rather than an established component of either research pattern (13).

### 7.1 Predominantly functional dysfunction pattern

The predominantly functional pattern describes presentations in which disease-specific evidence supports receptor, synaptic, or network dysfunction without demonstrated neuronal or tissue loss (2, 3); it is not a stage through which all disorders must pass and does not independently predict treatment responsiveness or reversibility. In neuronal surface-antibody syndromes, antibodies alter receptor trafficking through target-dependent mechanisms, induce receptor internalization, disrupt ligand–receptor interactions, or interfere with synaptic organization. Conventional MRI may remain normal despite clinically important functional impairment (27), but MRI negativity does not establish a predominantly functional pattern, and no validated clinical, imaging, antibody, or biomarker rule currently permits individual research-pattern assignment.

This predominantly functional pattern is relevant to cerebellar and connected brainstem phenotypes because Purkinje-cell circuits depend on precise temporal coding, inhibitory control, and synaptic plasticity (15, 19). [H] Target-dependent disruption of glutamatergic, GABAergic, glycinergic, or metabotropic signaling could alter Purkinje-cell output and connected infratentorial functions (15, 19), but no common downstream brainstem pattern has been validated across these disorders.

Across neuronal surface-antibody and immune-mediated cerebellar disorders, treatment response varies with the target, disease duration, relapse, and established structural injury (2, 3, 7, 15).

Candidate research features may include clinical fluctuation, serial absence or development of atrophy, and longitudinal neuronal-injury biomarkers; conventional MRI can be normal in anti-NMDAR encephalitis, cerebellar volume loss can evolve longitudinally in selected patients, and available cerebrospinal-fluid injury markers remain non-localizing and unvalidated as individual thresholds (27, 70, 71).

### 7.2 Disease-specific modifiers and hypothesis-generating mechanisms

The conditions under which functional synaptic dysfunction resolves, relapses, or accompanies structural injury remain uncertain and are likely to differ across disorders (2, 3, 7, 15).

Persistent inflammatory signaling may influence neuronal and circuit dysfunction. Neuronal pSTAT1 activation has been demonstrated in tissue from patients with intracellular-antigen-associated AE (72). [H] Whether interferon–STAT1 signaling has comparable relevance in neuronal-surface-antibody AE or cerebellar and brainstem subgroups requires separate prospective validation.

Blood–brain barrier and neurovascular-unit activation can increase CNS access by antibodies, complement components, cytokines, and lymphocytes; their contribution to selective cerebellum–brainstem injury requires direct study (2, 62).

[H] Experimental studies demonstrate complement-dependent synaptic tagging and microglial engulfment of synaptic material in non-AE neural systems (13, 73), providing a rationale for testing—but not establishing—inflammation-associated synaptic loss in cerebellar or brainstem AE.

Persistent antigenic stimulation is relevant to paraneoplastic syndromes, in which shared tumor–neural antigens sustain cytotoxic T-cell responses, and to relapsing neuronal surface-antibody disorders, in which renewed antibody production can reactivate synaptic dysfunction. These mechanisms should be analyzed separately because they involve different immune effectors and prognostic implications (2, 9, 34).

These processes are not mutually exclusive and should be modeled probabilistically rather than as a fixed transition sequence (2, 3, 7).

### 7.3 Established structural-injury pattern

The established structural-injury pattern describes presentations with disease-specific evidence of neuronal, axonal, or tissue injury, including neuropathological neuronal loss or longitudinally documented cerebellar or brainstem atrophy; it may occur without a preceding predominantly functional pattern and should not be interpreted as the inevitable endpoint of synaptic dysfunction. In presentations with independently documented structural injury, the clinical phenotype may reflect neuronal loss, axonal damage, or cerebellar and brainstem tissue injury in addition to any concurrent receptor or synaptic dysfunction (3, 9).

Paraneoplastic cerebellar degeneration, particularly anti-Yo/PCA1-associated disease, provides a clinicopathological example of an established structural-injury pattern characterized by Purkinje-cell-directed autoimmunity and cerebellar neuronal loss (67, 68). KLHL11 encephalitis provides another representative structural-injury phenotype with convergent imaging, neuropathological, and antigen-specific cellular evidence; the disease-specific data are reported once in Section 8.1 (8). In both settings, intracellular antibodies primarily function as diagnostic biomarkers, whereas T-cell-dominant neuronal injury may contribute to rapid progression, severe disability, and limited recovery after established neuronal loss (2, 3, 8–10, 68).

Candidate longitudinal features associated with structural injury include progressive deficits, evolving cerebellar or brainstem atrophy, neuronal-injury markers such as NfL, persistent CSF inflammation, and functional decline despite falling antibody titers; none has been validated for individual diagnosis, prognosis, treatment selection, or research-pattern assignment. In this pattern, antibody titers alone may not capture disease activity because established tissue injury and compartmentalized cellular inflammation can contribute independently to the clinical trajectory.

### 7.4 Imaging and biomarker correlates

The proposed research patterns do not replace the Graus clinical criteria for possible autoimmune encephalitis (1), antibody-specific diagnostic reasoning, PNS-Care criteria (9), or syndrome-specific criteria for BBE, MFS, GBS, ADEM, and MOGAD. Diagnostic classification and anatomical localization should precede and remain independent of research-pattern assignment. Current imaging studies demonstrate modality-specific abnormalities but do not validate a temporal transition between the two patterns or permit individual assignment. Biomarker interpretation is summarized in Section 8, whereas longitudinal infratentorial FDG-PET, diffusion, volumetric, and functional-connectivity measures and their prespecified technical safeguards are reviewed in Section 8.1 and **Table 3**.

**Table 3:** Current evidence and future validation approaches for the cerebellum–brainstem neuroimmune network.

Domain	Current evidence	Potential contribution to prespecified infratentorial evaluation	Current limitations and future directions
Clinical phenotyping	Cerebellar ataxia, ocular-motor abnormalities, vestibular symptoms, dysarthria, and brainstem syndromes are recognized manifestations across autoimmune encephalitis and related neuroimmune disorders.	Provides the clinical framework for identifying infratentorial immune-mediated phenotypes and defining patient subgroups.	Standardized phenotyping criteria specifically addressing cerebellar–brainstem involvement remain lacking. Prospective multicenter studies are required to establish reproducible clinical classifications.
Autoantibody profiling	Antibodies targeting neuronal surface antigens, intracellular antigens, and peripheral gangliosides are associated with heterogeneous cerebellar and brainstem manifestations.	Helps identify immune targets associated with different mechanisms, including synaptic dysfunction, immune-mediated neuronal injury, or peripheral–central overlap syndromes.	Antibody positivity alone does not fully determine anatomical involvement or clinical severity. Integrative analysis combining antibodies, immune profiles, and clinical trajectories is needed.
Cerebrospinal fluid (CSF) biomarkers	CSF inflammation, including pleocytosis, elevated protein levels, and intrathecal immune activation, may support immune-mediated neurological disease diagnosis.	Provides evidence of central immune activation and may complement antibody-based classification.	Conventional CSF markers lack specificity for cerebellar–brainstem involvement. Future studies should evaluate cellular and molecular immune signatures.
Conventional MRI	Brainstem or cerebellar abnormalities can be observed in selected patients, whereas normal MRI findings are also common.	Supports anatomical localization and exclusion of structural mimics.	Conventional MRI may remain normal despite clinically important autoimmune encephalitis, and currently reported abnormalities do not define a reproducible cerebellum–brainstem pattern. Serial structural imaging and prespecified quantitative approaches require prospective validation
FDG-PET	Current evidence comprises longitudinal single-case observations of cerebellar-vermis or brainstem hypermetabolism despite unrevealing structural MRI (Ances); a retrospective AE cohort showing cerebellar and brainstem hypermetabolism, with increased middle and upper brainstem metabolism associated with an unfavorable outcome (Dai); FDG-PET abnormalities in 47/55 patients compared with MRI abnormalities in 18/46 patients with available MRI, together with relative vermian hypermetabolism in 22/55 patients associated with cerebellar hypometabolism and atrophy (Liu); and dedicated brain FDG-PET/CT abnormalities in 52/61 patients in a single-center retrospective cohort (Probasco). In the publication-based synthesis by Kosek et al., FDG-PET abnormalities were reported in 93% and MRI abnormalities in 55% of 498 published cases from 234 reports. Approximately 38% of the 143 anti-NMDAR cases had abnormal cerebellar metabolism, while 16/19 patients with paraneoplastic cerebellar degeneration had cerebellar FDG-PET abnormalities, evenly divided between hypermetabolism and hypometabolism. Directional pontine and cerebellar data were not included in the main antibody-stratified heatmap because reference-region selection was seldom reported, and brainstem findings occurred in fewer than 5% of cases overall.	May provide complementary assessment of regional metabolic abnormalities when conventional MRI is unrevealing, but does not by itself establish cerebellum–brainstem network dysfunction, diagnostic specificity, prognosis, or individual research-pattern assignment.	Current evidence derives from longitudinal single-case observations, retrospective cohorts, and a publication-based synthesis; selection bias, unequal scan timing, treatment exposure, incomplete modality availability, verification bias, and reporting bias therefore limit inference. Frequencies derived from published cases should not be interpreted as population prevalence or validated diagnostic performance. Regional cerebellar or brainstem uptake should not be normalized to the same potentially affected structure under investigation. Kosek et al. illustrate this limitation because directional pontine and cerebellar findings could not be robustly interpreted when reference-region selection was inconsistently reported and were therefore excluded from the principal antibody-stratified heatmap. Studies should consequently prespecify an unaffected reference region, a validated data-driven normalization strategy, or absolute quantification. Dedicated brain acquisition should be distinguished from whole-body oncological PET. Because the vermis, medulla, and individual brainstem nuclei are small and CSF-adjacent relative to PET spatial resolution, partial-volume correction should be prespecified, with reporting of the PVC method, MRI acquisition and segmentation source, segmentation software and atlas, PET–MRI registration procedure, scanner-specific point-spread function, and sensitivity analyses with and without PVC. Structural atrophy and regional metabolism should be modeled jointly. Scan timing, recent seizures, sedation, treatment exposure, serum glucose, visual interpretation, and normative-database procedures should also be documented. No reproducible cerebellum–brainstem FDG-PET signature has yet been prospectively and systematically validated.
Diffusion MRI	Diffusion MRI studies in autoimmune encephalitis have demonstrated cerebral and cerebellar white-matter abnormalities, but prespecified tract-level evidence for the cerebellar peduncles and brainstem pathways remains limited.	May quantify tract-specific microstructural abnormalities in cerebellar peduncles and brainstem pathways as prospective research end points.	Conventional tensor-derived fractional anisotropy, mean diffusivity, radial diffusivity, and axial diffusivity are not fiber-specific in voxels containing crossing fibers or partial-volume effects. Multi-shell acquisition with constrained spherical deconvolution or fixel-based analysis should be preferred for tract-specific inference; when conventional DTI is used, crossing-fiber limitations should be stated explicitly.
Longitudinal volumetric MRI	Serial structural MRI can quantify longitudinal volume change, but validated cerebellar, vermian, pontine, or medullary thresholds for autoimmune encephalitis have not been established.	May quantify structural change in the cerebellar hemispheres, vermis, pons, medulla, hippocampal regions, and whole brain and relate these changes to disease burden and outcome.	Vermian, pontine, and medullary estimates are sensitive to image resolution, scanner or protocol changes, atlas choice, registration, and segmentation error. Studies should prespecify acquisition, segmentation software and atlas versions, manual quality control, scan–rescan or inter-rater reliability, and the minimum detectable volume change. Because MRI-derived segmentations may also serve as inputs to PET partial-volume correction, segmentation error can propagate into corrected regional uptake estimates; structural atrophy and FDG uptake should therefore be analyzed jointly.
Brainstem-optimized resting-state fMRI and connectomics	Distributed functional-connectivity abnormalities have been demonstrated in anti-NMDAR encephalitis, whereas cerebello-vestibular, ocular-motor, autonomic, and nucleus-level brainstem connectivity remain prespecified research targets rather than validated disease signatures.	May test nucleus- and network-level abnormalities within cerebellar–brainstem circuits and their associations with standardized clinical phenotypes.	Brainstem BOLD signals are affected by susceptibility distortion, cardiac and respiratory pulsatility, CSF movement, head motion, partial-volume effects, and lower temporal signal-to-noise ratio than many supratentorial regions. Susceptibility-distortion correction, cardiac and respiratory monitoring or physiological-noise modeling, rigorous motion control, nucleus-level registration or atlases, and regional temporal signal-to-noise ratio reporting should be prespecified.
Neurophysiological assessment	Abnormal ocular-motor function, vestibular abnormalities, and electrophysiological changes may reflect dysfunction of cerebellar and brainstem circuits.	Provides functional assessment of infratentorial pathway involvement.	Disease-specific sensitivity and prognostic value remain uncertain. Integration with imaging and immune biomarkers is needed.
Immune-cell profiling and cytokine analysis	Altered immune activation profiles have been described in autoimmune neurological disorders.	May help characterize immune states associated with different disease mechanisms.	Specific immune signatures linking peripheral immune responses with cerebellar–brainstem dysfunction remain undefined.
Spatial transcriptomics and single-cell approaches	Emerging technologies enable characterization of regional cellular and molecular alterations in neurological tissues.	May provide mechanistic insights into regional vulnerability and immune–neural interactions.	Current evidence is largely experimental. Translation into human autoimmune neurological disease remains a major challenge.
Longitudinal multimodal integration	Combination of clinical, immunological, imaging, and molecular datasets is increasingly feasible.	May enable development of predictive models for diagnosis, prognosis, and treatment response.	Prospective validation cohorts and harmonized analytical frameworks are required before clinical implementation.

**Table 3** summarizes currently available evidence and emerging approaches relevant to the cerebellum–brainstem network and the associated hypothesis-generating neuroimmune framework. Evidence levels vary across domains, and several approaches remain investigational rather than clinically validated. None currently defines a reproducible cerebellum–brainstem signature or supports individual diagnostic, prognostic, or research-pattern assignment. In the Kosek et al. publication-based synthesis, antibody-stratified directional hypermetabolic and hypometabolic data were not presented for the pons or cerebellum because these structures were frequently used as intracranial reference regions and reference-region selection was inconsistently reported.

### 7.5 Therapeutic implications and investigational rationale

When neuronal loss or structural cerebellar injury is independently documented in paraneoplastic or KLHL11-associated disease, functional recovery may be limited, although the degree of reversibility remains disease- and patient-specific (8, 67, 68). For patients with high-risk PNS phenotypes or high-risk antibodies, prompt phenotype- and antibody-guided cancer screening and appropriate oncological control are essential, with risk-adapted repeat screening considered when the initial evaluation is negative (9). Cerebellar or brainstem atrophy and neuronal-injury biomarkers should be evaluated as longitudinal prognostic research variables, but no validated threshold currently permits them to determine individual treatment decisions or irreversibility.

Treatment decisions should follow established AE-, antibody-, phenotype-, and PNS-specific principles (1, 9) rather than assignment to either research pattern. In anti-NMDAR encephalitis, earlier immunotherapy was associated with better outcome in an observational cohort (42), but this subtype-specific association neither validates research-pattern-based treatment assignment nor defines a universal therapeutic window. The framework identifies treatment timing, serial imaging, and neuronal-injury markers as variables for prospective testing within disease- and mechanism-defined cohorts.

## 8 Biomarkers and translational opportunities for prespecified cerebellar and brainstem research end points

This section evaluates biomarkers and research measures according to three questions: which biological process they measure, whether they localize injury, and whether they predict outcome. Current markers distinguish immune activity from tissue injury more readily than they localize cerebellar–brainstem involvement (70).

CSF pleocytosis, oligoclonal bands, intrathecal IgG synthesis, NfL, GFAP, cytokines, chemokines, and complement markers characterize inflammatory activity or tissue injury but do not localize pathology specifically to cerebellar or brainstem circuits (70).

Antibody profiling remains essential, but interpretation must distinguish directly pathogenic neuronal surface antibodies from intracellular antibodies that mainly function as biomarkers, and every positive result should be evaluated in relation to the clinical phenotype, CSF findings, assay characteristics, and oncological context (1–3, 9). In neuronal surface-antibody disorders, antibody titer, epitope specificity, IgG subclass, and intrathecal synthesis may be mechanistically informative because, in some disorders, these antibodies can alter receptor trafficking, ligand binding, ion-channel function, or synaptic organization (2, 3). In contrast, antibodies against intracellular or paraneoplastic antigens often serve primarily as biomarkers of a cytotoxic T-cell-mediated immune response rather than as direct effectors of neuronal injury (2, 3, 9). For this reason, antibody testing is most informative when integrated with clinical phenotype, CSF inflammation, tumor screening, neuroimaging, and longitudinal disability rather than interpreted in isolation (74–76).

Neuroimaging provides another layer of translational stratification. Dedicated brain FDG-PET currently has comparatively stronger, but still predominantly retrospective and methodologically heterogeneous, primary evidence for detecting metabolic abnormalities when routine MRI is unrevealing, whereas diffusion MRI, volumetry, rs-fMRI, and connectomic approaches remain investigational for cerebellum–brainstem localization (28, 33, 77, 78). For infratentorial hypothesis testing, imaging protocols should prespecify tract-level diffusion measures, longitudinal cerebellar–vermian–pontine–medullary volumetry, regional cerebellar and brainstem FDG uptake, and nucleus- or network-level functional connectivity, together with modality-specific acquisition, normalization, preprocessing, segmentation, quality-control, and reliability procedures, as detailed in Section 8.1 and **Table 3**. **Table 3** summarizes these research measures and their modality-specific technical requirements for longitudinal evaluation; none currently supports individual diagnostic, prognostic, treatment-selection, or research-pattern assignment.

Future studies should quantify B-cell, plasmablast/plasma-cell, T-helper, regulatory T-cell, CD8 T-cell, and tissue-resident memory T-cell signatures to test whether they distinguish predominantly humoral disease from cellular or compartmentalized inflammation. Immune-cell profiles should be analyzed separately in neuronal-surface-antibody AE, intracellular-antigen-associated PNS, glial or myelin-associated autoimmunity, and peripheral or overlapping anti-GQ1b syndromes, because these groups differ in anatomical compartment and dominant immune mechanism and should not be pooled without prespecified justification (2, 6, 9, 11, 32, 36). Their associations with relapse, treatment response, and structural outcome require prospective testing (70, 79).

Current AE management remains centered on established first-line and second-line immunotherapies; pathway-specific interventions beyond these approaches remain investigational and should not be incorporated into cerebellum–brainstem diagnostic or treatment algorithms without prospective, mechanism-defined validation (74).

### 8.1 Primary neuroimaging evidence: infratentorial findings and prospective validation

Current evidence does not show that a cerebellum–brainstem imaging signature is absent; rather, no reproducible infratentorial signature has yet been systematically sought using prospective, prespecified, and disease-stratified protocols, despite scattered primary observations of cerebellar, vermian, and brainstem abnormalities (8, 80–83). In a 53-patient anti-NMDAR encephalitis cohort, baseline MRI was normal in 28 patients (53%), whereas detected abnormalities were categorized as hippocampal, extrahippocampal, or combined; the study was not designed to test a prespecified infratentorial pattern (27). The six-patient anti-NMDAR encephalitis study by Leypoldt et al. identified a cerebral frontotemporal-to-occipital metabolic gradient, and other multimodal anti-NMDAR encephalitis studies demonstrated impaired hippocampal connectivity, widespread white-matter changes, hippocampal subfield atrophy, distributed resting-state network abnormalities, or longitudinal brain atrophy (28, 71, 77, 78, 84). However, these studies were designed around hippocampal, cerebral, or whole-brain outcomes rather than prespecified infratentorial regions and therefore did not constitute systematic tests of a cerebellum–brainstem imaging signature.

Direct primary observations indicate that infratentorial metabolic abnormalities can occur in autoimmune and related neuroimmune disorders, including cases in which metabolic changes were observed before overt structural abnormalities or clinically apparent progression; however, these observations remain limited by small sample sizes, retrospective designs, and heterogeneous disease populations. Ances et al. reported FDG hyperactivity in the cerebellar vermis approximately four months before the development of gait ataxia and brainstem FDG hyperactivity in an MRI-normal region before central hypoventilation developed (80). Dai et al. identified cerebellar and brainstem hypermetabolism as components of the acute-stage AE metabolic pattern and found that increased metabolism in the middle and upper brainstem was associated with unfavorable outcome (81). In a cohort of 55 patients with autoimmune cerebellar ataxia, Liu et al. reported FDG-PET abnormalities in 47/55 patients (85.5%), whereas MRI abnormalities were identified in 18/46 patients with interpretable MRI data (39.1%) (82). Relative vermian hypermetabolism was observed in 22/55 patients and was significantly associated with cerebellar hypometabolism, a higher cerebellar atrophy ratio, and lower CSF white-cell counts (82). Because this was a relative finding in a cohort with cerebellar hypometabolism and atrophy, its interpretation is sensitive to the normalization strategy and to differential partial-volume effects in the thin, CSF-adjacent vermis. Establishing a biological vermis–hemisphere dissociation would require dedicated brain acquisition, an unaffected or validated data-driven reference, prespecified partial-volume correction with MRI-based segmentation and PET–MRI registration, and longitudinal replication (82, 85–88). Kosek et al. synthesized patient-level findings from 498 published cases across 234 reports and found abnormal FDG-PET findings in 93% and MRI abnormalities in 55% of the published cases (83). Because the sampling frame consisted of publications reporting brain FDG-PET findings, it was likely enriched for abnormal or otherwise reportable PET patterns; these proportions should therefore be interpreted as distributions among published cases rather than as population prevalence or validated diagnostic performance. Importantly, Kosek et al. did not include directional pontine or cerebellar findings in the main antibody-stratified hypermetabolism–hypometabolism heatmap because the pons and cerebellum were commonly used as intracranial reference regions and reference-region selection was seldom reported, precluding robust interpretation of metabolic direction in these structures (83). Nevertheless, abnormal cerebellar metabolism was reported in approximately 38% of the 143 published anti-NMDAR cases, although reliable separation into hypermetabolic and hypometabolic categories was not available (83). In the separate subgroup of 19 patients with paraneoplastic cerebellar degeneration, 16/19 (84%) had cerebellar FDG-PET abnormalities, comprising hypermetabolism in 8/19 and hypometabolism in 8/19, whereas MRI was abnormal in 3/16 scanned patients, all of whom had cerebellar atrophy (83). Reported brainstem findings occurred in fewer than 5% of cases overall and were therefore confined to the individual-level supplementary dataset rather than included in the principal tables or figures (83). These publication-derived findings should be interpreted descriptively because publication and selection bias, incomplete reporting of scan timing and treatment exposure, variable acquisition and interpretation methods, and especially unreported reference-region selection prevent estimation of the population prevalence of cerebellar or brainstem metabolic abnormalities or validation of a reproducible infratentorial signature (83).

Whereas the preceding FDG-PET studies primarily demonstrate functional or metabolic abnormalities, KLHL11 encephalitis provides complementary MRI-visible and clinicopathological evidence of structural injury. In the retrospective tertiary-care KLHL11-IgG cohort, brain MRI at diagnosis was available in 37 of 39 patients; 7/37 had no detected abnormality, whereas 28/37 (76%) had T2/FLAIR hyperintensity involving the temporal lobe (n = 12), cerebellum (n = 9), brainstem (n = 3), or diencephalon (n = 3) (8). Because temporal-lobe and diencephalic abnormalities were also represented, the imaging distribution was not exclusively infratentorial (8). A cerebellar biopsy and two autopsies demonstrated Purkinje-cell loss and Bergmann gliosis, while KLHL11 exposure increased CD69-expressing CD4+ and CD8+ T cells in patient-derived cultures compared with healthy controls (8). Together, these imaging, neuropathological, and antigen-specific cellular findings support a T-cell-linked structural-injury phenotype, although the retrospective tertiary-care design and incomplete MRI availability limit generalizability.

Dedicated brain FDG-PET has comparatively stronger—but still predominantly retrospective and methodologically heterogeneous—evidence for detecting metabolic abnormalities when routine MRI is unrevealing (33, 80–83); however, its diagnostic, prognostic, treatment-selection, and individual research-pattern-assignment value for cerebellar or brainstem immune involvement remains unvalidated. In the single-center retrospective study by Probasco et al., dedicated brain FDG-PET/CT was abnormal in 52 of 61 patients (85%), compared with initial MRI in 23 of 57 (40%), EEG in 17 of 56 (30%), and CSF inflammation in 34 of 55 (62%) (33). FDG-PET was performed at a median of four weeks after symptom onset and a median of four days after MRI, whereas the comparison used initial MRI, EEG, and CSF findings; the observed difference therefore reflects both modality and unequal testing time and is susceptible to spectrum and verification bias. Moreover, these abnormality-detection rates were obtained using dedicated brain acquisition, three-dimensional stereotactic surface projection, and an age-matched control database and should not be extrapolated to routine whole-body oncological PET without equivalent brain acquisition and quantitative analysis.

Prospective infratentorial imaging protocols should also prespecify modality-specific technical safeguards. For FDG-PET, regional cerebellar or brainstem uptake should not be normalized to the same potentially affected structure under investigation. This limitation is directly illustrated by Kosek et al., who excluded directional pontine and cerebellar findings from their principal antibody-stratified heatmap because these structures were commonly used as intracranial reference regions and reference-region selection was seldom reported (83). Prospective studies should therefore prespecify an alternative unaffected reference region, validated data-driven normalization, or absolute quantification; distinguish dedicated brain acquisition from whole-body oncological PET; and document scan timing, recent seizures, sedation, treatment exposure, serum glucose, visual interpretation, and normative-database procedures (33, 83, 85). Because the vermis, medulla, and individual brainstem nuclei are small and CSF-adjacent relative to PET spatial resolution, regional analyses should prespecify partial-volume correction and report the PVC method, MRI segmentation source and atlas, PET–MRI registration procedure, scanner-specific point-spread function, and sensitivity analyses performed with and without PVC (86–88). Structural atrophy should be modeled jointly with regional metabolism because differential tissue loss can bias apparent uptake and may otherwise be misinterpreted as a biological metabolic difference (86–88). For diffusion MRI, conventional tensor-derived FA, MD, RD, and AD are not fiber-specific in voxels containing crossing fibers or partial-volume effects, particularly in pontine and cerebellar-peduncle regions; multi-shell acquisition with constrained spherical deconvolution or fixel-based analysis should therefore be preferred for tract-specific inference, and crossing-fiber limitations should be stated explicitly when conventional DTI is used (89, 90). Brainstem resting-state fMRI should include susceptibility-distortion correction, cardiac and respiratory monitoring or physiological-noise modeling, rigorous motion control, nucleus-level anatomical registration or atlases, and reporting of regional temporal signal-to-noise ratio (91–93). Longitudinal volumetry should prespecify image resolution, scanner and acquisition consistency, segmentation software and atlas, manual quality control, scan–rescan or inter-rater reliability, and the minimum detectable volume change for the vermis, pons, and medulla before small differences are interpreted as disease progression (94–97).

The appropriate conclusion is therefore not that a cerebellum–brainstem imaging signature has failed to emerge, but that it has not yet been systematically sought using prospective, prespecified, and disease- and mechanism-stratified protocols. Existing primary observations are compatible with clinically meaningful metabolic or structural involvement of the vermis, cerebellar hemispheres, and brainstem (8, 80–83), but heterogeneity in diagnosis, scan timing, treatment exposure, normalization, acquisition, and analysis precludes designation of a validated diagnostic, prognostic, treatment-selection, or research-pattern-assignment biomarker. Prospective multicenter studies should test these observations using standardized infratentorial end points, prespecified analytical procedures, disease- and mechanism-stratified cohorts, and independent replication.

## 9 Discussion

The cerebellum–brainstem network is used in this Review as an anatomical and clinical domain, whereas the cerebellum–brainstem neuroimmune framework is a hypothesis-generating method for organizing disease-specific evidence rather than a newly defined disease entity or validated pathogenic pathway. By synthesizing evidence from antibody-mediated synaptic dysfunction, cellular immune injury, neuroimaging, and biomarker studies, this framework highlights how heterogeneous immune mechanisms may converge on shared infratentorial clinical phenotypes. However, substantial uncertainties remain regarding the prevalence, regional specificity, temporal evolution, and therapeutic implications of cerebellar and brainstem involvement across autoimmune encephalitis and related neuroimmune disorders. The following sections discuss the major limitations of current evidence and outline priorities required for prospective validation.

### 9.1 Limitations and knowledge gaps

Several limitations warrant consideration when interpreting the cerebellum–brainstem neuroimmune framework. First, the frequency of direct cerebellar and brainstem involvement in AE remains uncertain because most diagnostic frameworks and available cohorts were not designed for standardized infratentorial phenotyping. This should be regarded as an evidence gap rather than as a quantified estimate of under-recognition (1, 3).

Second, the evidence base is heterogeneous. Well-characterized syndromes such as anti-NMDAR encephalitis are supported by cohort and experimental data, whereas rare antibody-associated cerebellar or brainstem syndromes rely mainly on case reports or small series. Selected rare antibody-associated disorders, including Homer-3, EEF1D, and KLHL11 autoimmunity, together with seronegative immune-mediated cerebellar ataxias, provide mechanistic and clinicopathological insights (8, 61, 98, 99). However, their evidence levels differ from those of better-characterized AE syndromes and require cautious interpretation.

Third, shared symptoms or antibody positivity do not establish a common nosological category: MFS and GBS remain predominantly peripheral neuropathies, BBE is a central brainstem syndrome, and only explicitly documented overlap cases should be described as involving both compartments (11, 12, 14). ADEM and MOGAD may include brainstem or cerebellar involvement but remain distinct acquired demyelinating CNS disorders and should not be pooled mechanistically with neuronal-surface-antibody AE (5, 6). Primary viral encephalitis is not included as core evidence because direct viral neurotropism, antiviral immune responses, and host genetic susceptibility differ fundamentally from antibody-mediated receptor internalization, ganglioside-associated peripheral nerve injury, and intracellular-antigen-associated cellular autoimmunity (100); any viral-model extrapolation is therefore labeled [H]. This is especially important for intracellular antigens and paraneoplastic neurological syndromes, in which antibodies often serve as diagnostic biomarkers of a broader T-cell-mediated immune response rather than direct mediators of neuronal injury. Negative antibody testing does not exclude immune-mediated disease, but diagnostic reasoning must remain syndrome-specific: possible AE should be assessed using the Graus clinical criteria (1), BBE using central brainstem clinical criteria (12), and MFS or GBS using peripheral neuropathy classifications rather than an AE framework (11).

Fourth, existing imaging studies are heterogeneous in modality, scan timing, treatment exposure, disease severity, and disease subtype, and no cerebellum–brainstem-specific diagnostic, prognostic, treatment-selection, or research-pattern-assignment signature has been independently validated.

Fifth, available fluid biomarkers reflect general immune activity or tissue injury rather than regional localization, so their interpretation requires integration with phenotype, antibody profile, imaging, and longitudinal course (70).

Sixth, pediatric and adult presentations should not be overcombined because children differ from adults in clinical presentation, differential diagnosis, paraclinical findings, antibody profiles, treatment response, and long-term outcome (101). Pediatric assessment should give separate consideration to ADEM, MOGAD, OMAS, developmental phenotypes, and age-specific mimics (5, 6, 22), whereas adult-onset cerebellar or brainstem syndromes require greater attention to antibody-defined disease and paraneoplastic risk (9). Pediatric and adult biomarker and imaging data should be analyzed separately or with prespecified age adjustment because age affects baseline neuronal-injury biomarker concentrations and structural brain measurements (97, 102), while pediatric AE also differs from adult AE in clinical presentation, differential diagnosis, antibody profiles, treatment response, and long-term outcome (101).

### 9.2 Future directions

Future studies should validate four predefined domains: standardized infratentorial phenotyping, longitudinal multimodal imaging, integrated fluid and cellular biomarkers, and mechanism-stratified treatment response. Multicenter cohorts should record ataxia, ocular-motor dysfunction, dysarthria, dysphagia, respiratory disturbance, sleep dysregulation, autonomic instability, and altered consciousness using harmonized assessments.

Longitudinal biomarker studies should test the relationships among functional dysfunction, structural injury, relapse, and outcome without presuming a validated transition or biomarker-defined boundary between the two research patterns. Serial CSF and serum NfL, GFAP, cytokines, chemokines, complement markers, antibody titers, epitope specificity, IgG subclass, and B- and T-cell signatures should be analyzed alongside clinical outcomes and imaging trajectories. These longitudinal analyses should prospectively test whether multimodal biomarker combinations predict relapse, treatment response, and structural outcomes within disease- and mechanism-defined cohorts.

Prospective imaging studies in AE and related neuroimmune disorders should use standardized longitudinal multimodal acquisition within separately analyzed disease- and mechanism-defined cohorts, with structural MRI and, where clinically justified, dedicated brain FDG-PET, diffusion MRI, and resting-state fMRI obtained at prespecified clinical time points. Prespecified infratentorial research end points should include regional structural MRI abnormalities, dedicated-brain FDG-PET measures, tract-specific diffusion measures that address crossing-fiber limitations, longitudinal cerebellar and brainstem volumetry with age- and normalization-aware analysis, and nucleus- or network-level functional connectivity (8, 27, 28, 33, 89, 90, 97). Data-driven or machine-learning analyses should be trained and externally validated in independent disease- and mechanism-defined cohorts and related to standardized phenotypes, treatment exposure, and longitudinal outcomes before being considered clinically interpretable biomarkers.

Mechanistic studies should investigate how immune effector pathways interact with region-specific cerebellar and brainstem vulnerability. Single-cell and spatial transcriptomics, human neuron–glia–vascular models, organoids, and post-mortem tissue studies should be used to determine which neuronal, glial, vascular, and immune-cell populations contribute to local cerebellar and brainstem injury in AE. Future studies should separately test blood–brain barrier activation, glial and complement-dependent synaptic responses [H], interferon–STAT1 signaling in intracellular-antigen-associated AE, and compartmentalized CNS inflammation rather than treat these processes as an established unified pathway in cerebellum–brainstem AE (13, 35, 72).

Therapeutic studies should evaluate treatment timing prospectively within disease- and mechanism-defined cohorts rather than infer a universal therapeutic window from retrospective associations. Future studies should analyze central AE, BBE, PNS, ADEM, MOGAD, peripheral MFS or GBS phenotypes, and explicitly defined central–peripheral overlap syndromes as separate disease groups before testing whether any findings can legitimately be integrated within the broader cerebellum–brainstem framework (1, 5, 6, 9, 11, 12). Prospective trials and registries should first evaluate established disease-specific immunotherapy, relapse prevention, and tumor control where relevant (9, 74), whereas pathway-specific interventions should be tested separately in mechanistically defined disease subgroups.

Together, these studies will determine whether the cerebellum–brainstem framework has reproducible diagnostic, prognostic, or therapeutic value.

## 10 Conclusions

The cerebellum–brainstem network provides an anatomical–clinical domain within which the neuroimmune framework organizes disease-specific evidence concerning antibody-mediated dysfunction, cellular immune injury, and cerebellar synaptopathy, without assuming that their relationships to individual infratentorial phenotypes have been fully validated. Its contribution is integrative: it distinguishes direct regional injury from secondary network and peripheral mechanisms, preserves the nosological boundary between AE, BBE, and MFS/GBS, and defines testable imaging and biomarker questions. The circuit hypotheses and two non-sequential research patterns remain hypothesis-generating tools rather than validated pathogenic pathways, clinical stages, biomarker-defined transitions, treatment windows, or therapeutic algorithms.
